# Interpreting terminal selectivity in undirected C(sp^3^)–H functionalization: kinetic insights and mechanistic implications

**DOI:** 10.1039/d6sc02478e

**Published:** 2026-06-16

**Authors:** Jia-Lin Tu, Lin Guo, Binbin Huang, Wujiong Xia

**Affiliations:** a State Key Lab of Urban Water Resource and Environment, Harbin Institute of Technology (Shenzhen) Shenzhen 518055 China xiawj@hit.edu.cn; b Faculty of Arts and Sciences, Beijing Normal University Zhuhai 519085 China binbinhuang@bnu.edu.cn; c School of Chemistry and Chemical Engineering, Henan Normal University Xinxiang Henan 453007 China

## Abstract

Undirected terminal C(sp^3^)–H functionalization represents a fundamental challenge in organic synthesis. Although early transition-metal-, enzyme-, and zeolite-based platforms have achieved high terminal selectivity through well-defined steric control mechanisms, a unifying mechanistic framework remains elusive for rationalizing such selectivity in the rapidly expanding radical-mediated C(sp^3^)–H functionalization manifold. This review introduces a stepwise kinetic analysis that deconstructs the origins of terminal selectivity into two major kinetic regimes: (1) “Front-end Attack Control”, wherein selectivity is predominantly governed by the initial C–H bond cleavage event; and (2) “Back-end Capture Control”, which arises during the functionalization step following non-selective, reversible C–H bond cleavage. Furthermore, for systems that transcend this binary classification, we further examine “synergistic control” in multi-step relay catalysis and the “intermediate regime”, wherein selectivity emerges from combined kinetic and thermodynamic effects. Through systematic analysis of diverse catalytic platforms and representative case studies, we aim to elucidate the mechanistic origins of site selectivity and establish a theoretical foundation for the rational design of precision C(sp^3^)–H functionalization systems, thereby facilitating the transition from *post hoc* rationalization to predictive catalyst design.

## Abstract 2

In this review, we introduce a stepwise kinetic analysis to dissect the origins of terminal selectivity observed in undirected C(sp^3^)–H functionalization.

## Graphical abstract

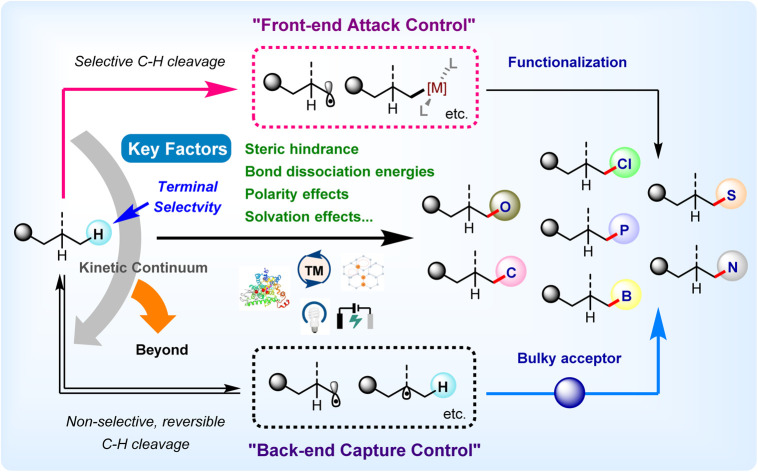

## 1. Introduction

Direct and selective functionalization of ubiquitous C(sp^3^)–H bonds represents one of the most transformative frontiers in modern organic synthesis.^1,2^ This approach aims to bypass traditional prefunctionalization steps, enabling the construction of complex molecules with maximal atom- and step-economy. However, a central challenge in this field is achieving precise site selectivity among multiple, chemically similar C–H bonds. Historically, the reactivity has been viewed through two distinct lenses. In radical-mediated processes, reactivity is typically governed by the bond dissociation energy (BDE) of C–H bonds (3° < 2° < 1°), resulting in thermodynamic preference for functionalization at more substituted, internal positions.^3^ In contrast, in organometallic C–H activation (*e.g.*, mediated by Ir, Rh, or W), the formation of metal–alkyl intermediates often favors terminal sites due to steric constraints or the greater thermodynamic stability of metal–primary carbon bonds (M-1° > M-2° > M-3°).^4^ Despite these well-established principles, the rapidly evolving field of catalytic C(sp^3^)–H functionalization—particularly involving photochemical and electrochemical methods—exhibits complex selectivity patterns that cannot be fully explained by conventional thermodynamic or steric models.^5^ While recent reviews have comprehensively summarized strategies for C(sp^3^)–H functionalization enabled by directing groups, particularly those employing 3d transition-metals,^6^ a systematic analysis of undirected terminal functionalization remains lacking.

**Fig. 1:**
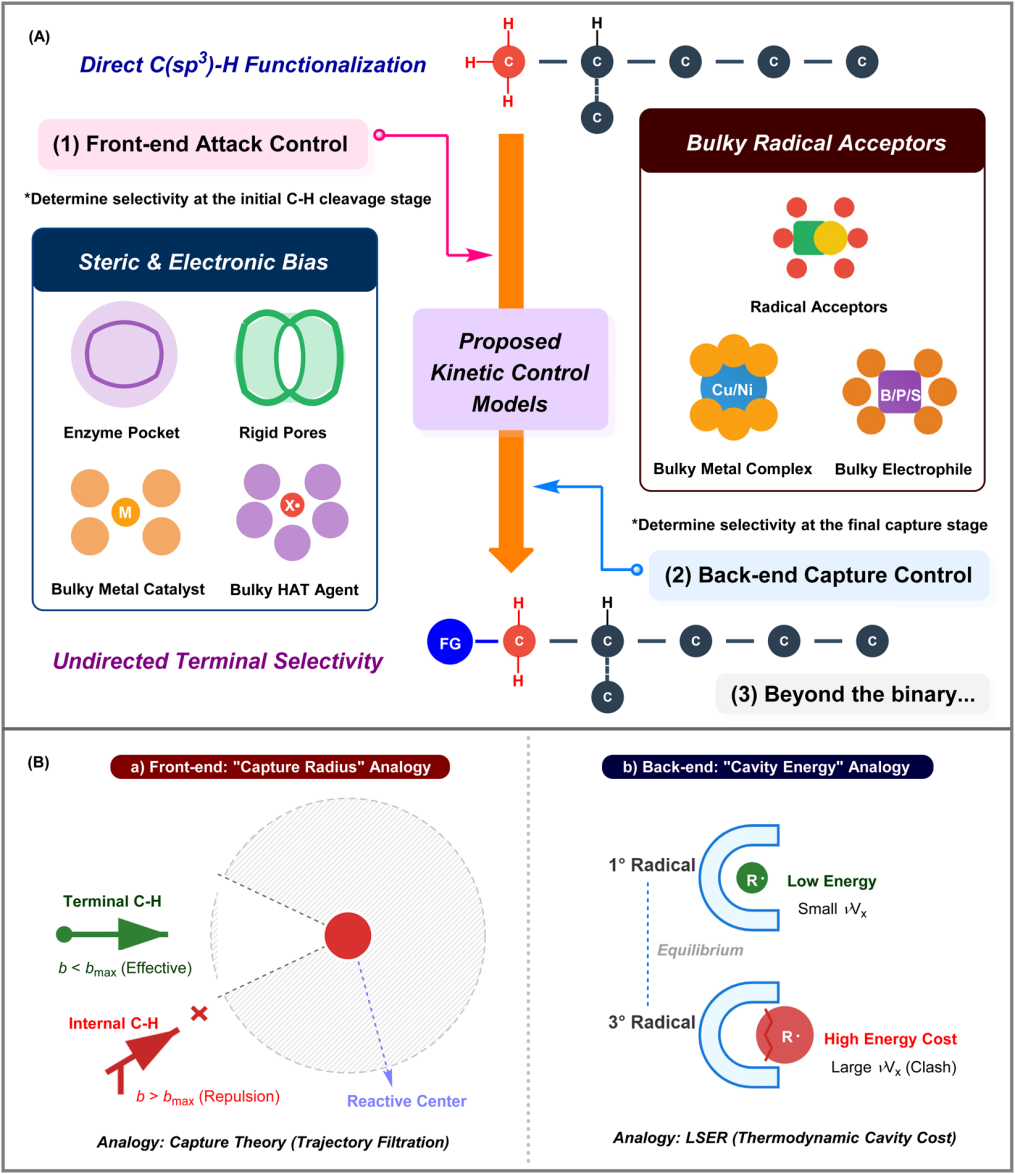
Interpretation of terminal selectivity in undirected C(sp^3^)–H functionalization. (A) Two fundamental kinetic control models proposed for achieving terminal C(sp^3^)–H selectivity. (B) Physicochemical analogies for the two major kinetic control models. ^*a*^The hatched area represents the repulsive field (steric or electronic) created by the catalyst; only substrates capable of entering the sterically defined “trajectory cone” (*b* < *b*_max_) can access the central reactive center, while bulky internal sites are deflected. ^*b*^The trapping agent creates a rigid micro-environment. The prohibitive cavity formation penalty (*v*V_x_) for large internal radicals translates into a high kinetic barrier (Δ*G*‡), preventing capture and forcing the reaction back into the reversible hydrogen atom transfer cycle.

This review analyzes terminal selectivity in C(sp^3^)–H functionalization reactions, grounded in the kinetic barriers associated with distinct stages of the reaction pathway. These kinetic barriers often disfavor terminal sites, which are thermodynamically less stable, consistent with the Bell–Evans–Polanyi principle.^7^ To rationalize the diverse experimental observations, we initially categorize these reactions into two archetypal modes based on the primary stage at which terminal selectivity is determined (Fig. 1A): (1) “Front-end Attack Control”, wherein site selectivity is mainly established during the initial C(sp^3^)–H bond cleavage step, leading preferentially to terminal intermediates—this can be achieved through steric confinement in enzyme pockets,^8^ rigid porous materials,^9^ or bulky transition-metal catalysts;^10^ and (2) “Back-end Capture Control”, wherein the initial C–H activation is non-selective and reversible, generating a mixture of radical or organometallic intermediates, with selectivity imposed in a subsequent trapping event—such as through the use of sterically demanding radical acceptors.^11^ To provide theoretical support for this framework, conceptual analogies are drawn to Capture Theory^12–14^ and Linear Solvation Energy Relationships (LSER)^15–18^ (Fig. 1B). It should be emphasized that these analogies serve as qualitative heuristics to enhance physicochemical understanding; they are not intended as quantitative, first-principles derivations from gas-phase collision theory or bulk solvation models to the condensed-phase catalytic potential energy surface.

Although this binary framework provides a robust conceptual anchor, we acknowledge that many contemporary catalytic systems operate within an increasingly nuanced mechanistic landscape, which often transcends these idealized categories. Accordingly, we further discuss how the interplay between different factors gives rise to synergistic and intermediate control modes. This expanded framework, encompassing “Front-end”, “Back-end” and beyond, delivers a unified mechanistic interpretation for terminal selectivity in undirected C(sp^3^)–H functionalization, with an aim to lay a theoretical basis for the rational design of next-generation catalytic systems.

## 2. Interpreting selectivity factors using HAT as a model system

While organometallic C–H activation follows distinct mechanistic principles, which is often governed by metal–carbon bond strengths, the hydrogen atom transfer (HAT) manifold provides a more intuitive basis for elucidating the interplay among BDEs, steric hindrance, polar effects, and solvent effects. Within this framework, kinetic behavior can be effectively modeled using a Marcus-theory-type approach derived from the cross relation,^19,20^ thereby offering a robust platform for analyzing reactivity patterns. A systematic analysis of these contributing factors within the HAT paradigm enables a fundamental understanding of “Front-end” and “Back-end” selectivity, a concept that would extend to a broader range of catalytic transformations.

### 2.1 Fundamental elements and selectivity principles of HAT reactions

**Fig. 2:**
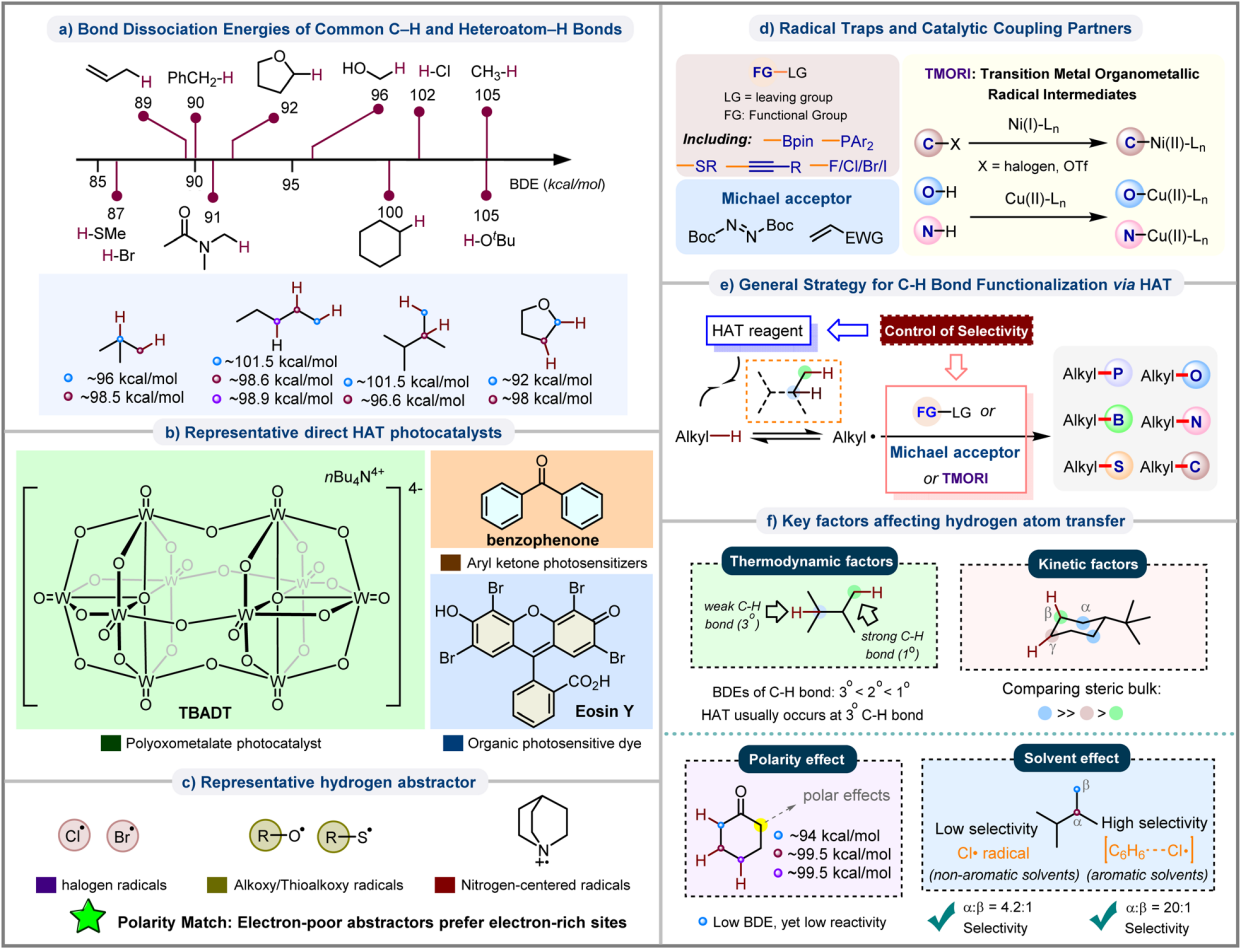
An overview of hydrogen atom transfer (HAT) pathways for C(sp^3^)–H bond functionalization. (a) Bond dissociation energies of common C–H and heteroatom–H bonds. (b) Representative direct HAT photocatalysts. (c) Representative hydrogen abstractors. (d) Radical traps and catalytic coupling partners. (e) General strategy for C–H bond functionalization *via* HAT. (f) Key factors affecting hydrogen atom transfer.

A comprehensive understanding of HAT reaction selectivity requires careful examination of the key elements governing the reaction pathway (Fig. 2). From a thermodynamic standpoint, the driving force for HAT processes originates from the energy difference between bond formation and bond cleavage (Fig. 2a). Thermodynamically favorable HAT events occur when the newly formed X–H bond is more stable than the cleaved C–H bond. For instance, tertiary C(sp^3^)–H bonds exhibit BDEs of approximately 96 kcal mol^−1^, whereas primary C(sp^3^)–H bonds have significantly higher BDEs of ∼101.5 kcal mol^−1^.^21,22^ Within the HAT manifold, this energetic hierarchy implies that reactions under thermodynamic control preferentially target weaker tertiary C–H sites. The initiation of HAT reactions requires the generation of highly reactive species, typically achieved through photocatalytic activation.^23^ Such reactive intermediates may include the photoexcited state of a photocatalyst, such as tetrabutylammonium decatungstate (TBADT) or the organic dye Eosin Y (Fig. 2b).^24^ Alternatively, active HAT reagents can be generated *in situ*, with common examples including halogen, oxygen-, and sulfur-centered radicals (Fig. 2c).^25–27^ Following hydrogen atom abstraction and the formation of alkyl radicals, these transient intermediates must be efficiently captured by radical acceptors or catalytic coupling partners to yield the desired products (Fig. 2d).^1,2,24,28^ This sequence constitutes the general strategy for C–H functionalization *via* HAT (Fig. 2e).

It is well established that the regioselectivity of HAT reactions is governed by four principal factors (Fig. 2f): (1) thermodynamic control, which arises from differences in C–H bond dissociation energies, leading to preferential reactivity at positions with the lowest BDE (3° < 2° < 1°); (2) kinetic control, mediated by steric effects,^29^ whereby bulky reagents selectively access less hindered C–H bonds; (3) polar effects, reflecting electronic compatibility between reagents and substrates—electrophilic radicals tend to react with electron-rich C–H bonds, occasionally overriding thermodynamic preferences;^30,31^ and (4) solvent effects, which influence both reactivity and selectivity through differential stabilization of transition states and solvation of reactive intermediates.^32–34^ The complex interplay among these factors ultimately determines the observed selectivity in HAT processes. Although these parameters provide a foundational understanding, they are not always sufficient to predict outcomes in intricate catalytic systems. A more predictive model may be achieved by examining the dynamic interplay among thermodynamics, steric constraints, electronic effects, and solvent effects. Particularly in relation to kinetic bottlenecks.

### 2.2 Case study I: synergy of factors in decatungstate photocatalysis

**Fig. 3:**
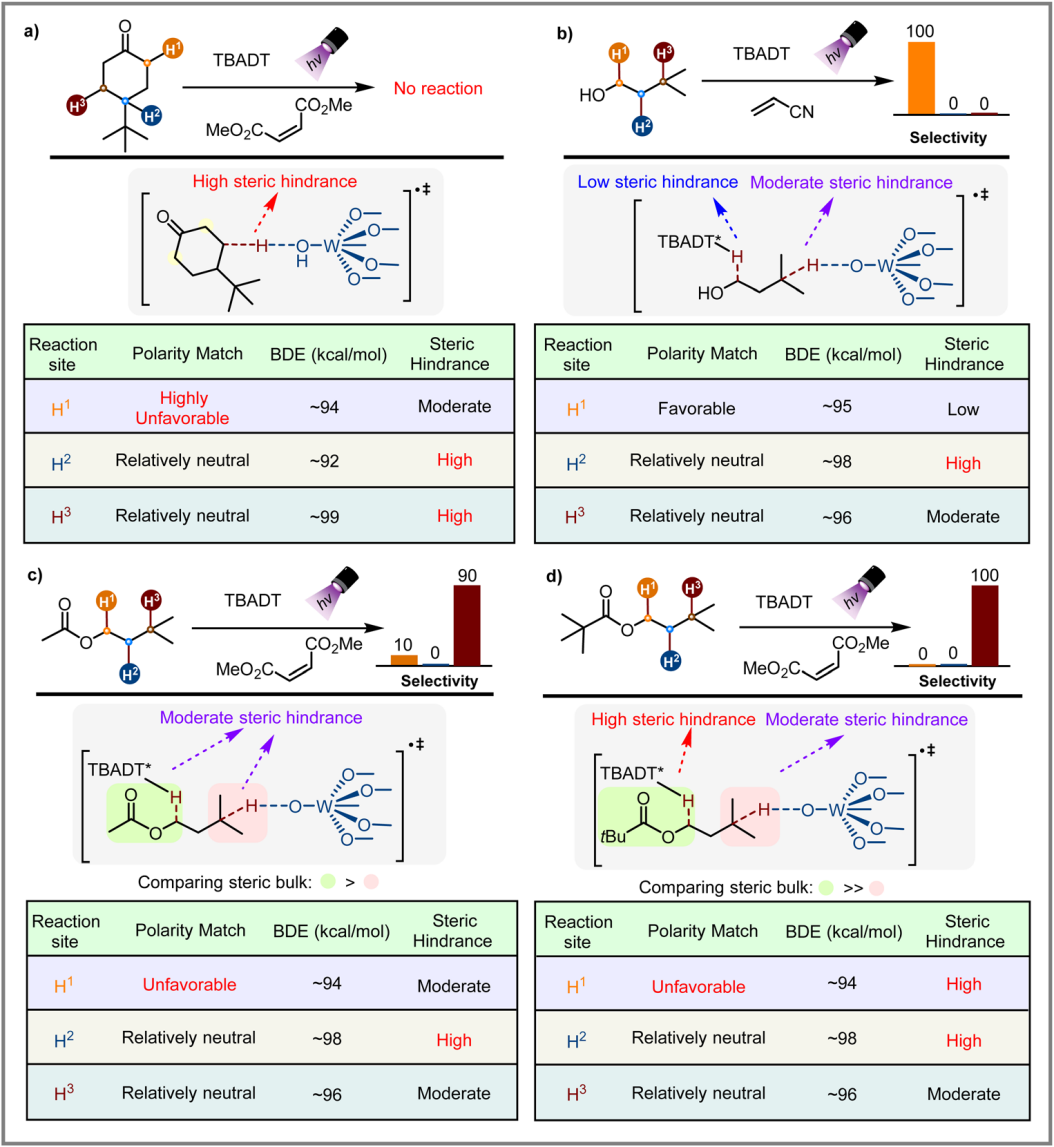
Interplay of BDEs, polar effects, and steric hindrance in TBADT-photocatalyzed C–H activation. (a) Ketone substrate rendering the α-C–H bond unreactive due to polar mismatch. (b) Alcohol substrate displaying optimal electronic alignment and minimal steric hindrance. (c) Methyl ester substrate illustrating a nuanced competition with minor α-reactivity. (d) *tert*-Butyl ester substrate demonstrating complete suppression of the α-pathway *via* increased steric bulk.

The photocatalyst TBADT serves as an exemplary system for dissecting the intricate interplay among BDEs, polar effects, and steric factors, as the reactivity of its excited state (*wO) is characterized by a distinctive duality: strong electrophilicity arising from the electronic hole, coupled with significant steric hindrance imposed by the bulky polyoxometalate framework.^35–37^ As illustrated in Fig. 3, the overall reaction outcome is governed by a series of multifactorial trade-offs. Polar effects can exert a decisive “veto” on reactivity: despite a favorable BDE (∼94 kcal mol^−1^), the α-C–H bond adjacent to a ketone is rendered unreactive toward the electrophilic *wO species, as the electron-withdrawing carbonyl group destabilizes the charge-transfer transition state (Fig. 3a). In contrast, the alcohol exhibits optimal synergy: favorable BDE (∼95 kcal mol^−1^), minimal steric hindrance, and alignment in polarity, leading to exclusive α-selectivity (Fig. 3b). Esters illustrate a more nuanced competition: while the methyl ester permits minor α-reactivity (10%), increasing steric bulk to a *tert*-butyl group synergizes with inherent polar mismatch to completely suppress the α-pathway, thereby shifting selectivity entirely toward the thermodynamically favored product (Fig. 3c and d).

These trade-offs illustrate how TBADT acts as a strict “electronic filter” at the hydrogen abstraction stage, a hallmark of what we classify as “Front-end Attack Control” (see Section 2.4).

### 2.3 Case study II: the “adamantane paradox”

The “adamantane paradox” refers to the elusive regioselectivity observed between two distinct types of C(sp^3^)–H bonds in adamantane, a phenomenon rooted in the exceptional structural rigidity of the adamantane scaffold compared to conventional linear or monocyclic alkanes.^38^ This rigidity introduces a fundamental dichotomy in HAT processes: although the electron-rich bridgehead C–H bonds are kinetically accessible to electrophilic reagents due to favorable electronic alignment, their activation is thermodynamically disfavored because the resulting carbon-centered radical cannot adopt a planar, stabilized geometry.^39,40^ This intrinsic conflict between kinetic preference and thermodynamic penalty renders conventional static models inadequate, leading to the contradictory selectivity outcomes and mechanistic interpretations observed in current literature.

**Fig. 4:**
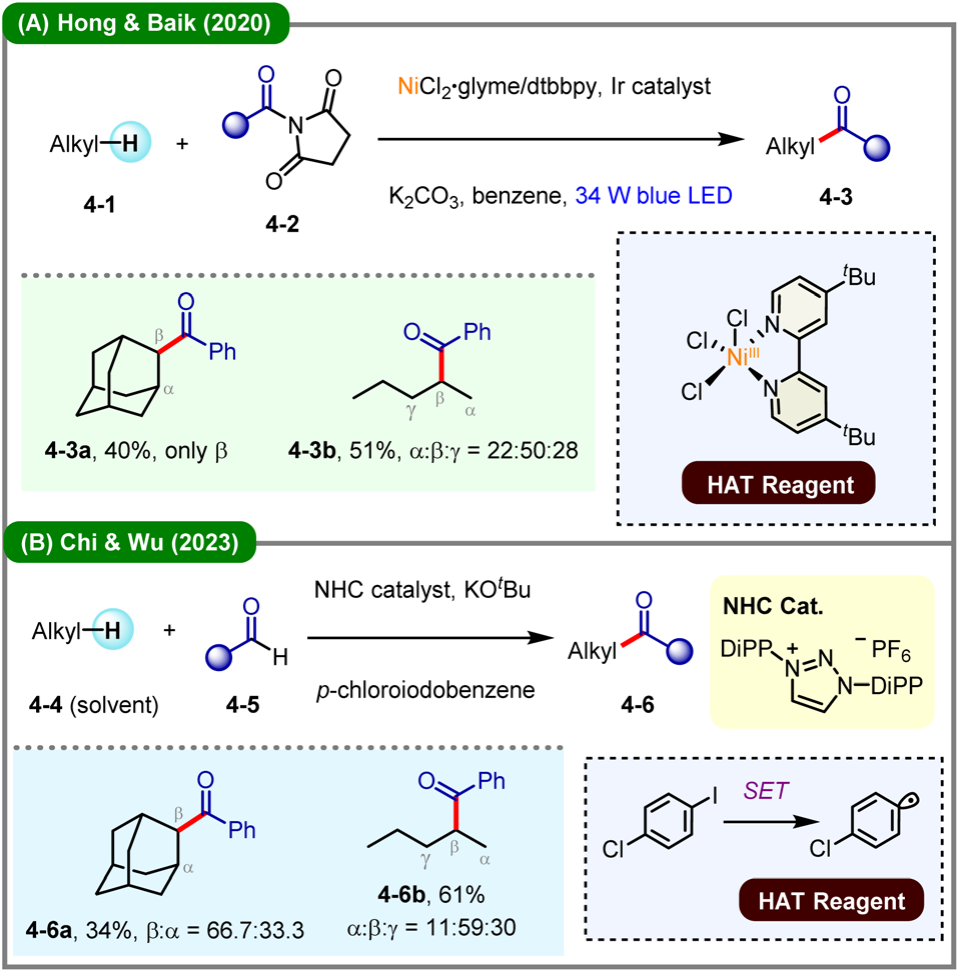
Contrasting selectivity on rigid (adamantane) and flexible alkanes. (A) Hong and Baik's work; (B) Chi and Wu's work.

The Ir/Ni dual catalytic system developed by Hong and Baik achieves exclusive β-selectivity (4-3a), which is a rare regioselective outcome in radical-mediated adamantane C–H functionalization reactions (Fig. 4A).^39^ This unique selectivity can be well rationalized by the “Front-end Attack Control” model: the *in situ* generated Ni(iii) species with substantial steric hindrance kinetically precludes the activation of the sterically congested bridgehead α-C–H bond. In comparison, the NHC-organocatalyzed system reported by Chi and Wu delivers a moderate β-selectivity (4-6a, *β* : *α* ≈ 2 : 1), which employs an aryl radical generated *via* single-electron reduction of *p*-chloroiodobenzene as the HAT reagent (Fig. 4B).^40,41^ The unconventional regioselectivity observed in this protocol is ascribed by the authors to the thermodynamic instability of the α-radical intermediate. Specifically, the rigid cage skeleton of adamantane imposes severe geometric constraints on the bridgehead α-radical, rendering this radical species energetically unfavorable and thus disfavoring α-functionalization. Crucially, however, this mode of regiocontrol fails completely when applied to flexible linear alkanes (*e.g.*, 4-3b and 4-6b), underscoring that the observed site preference is intrinsically linked to the unique structural architecture of the adamantane framework.

**Fig. 5:**
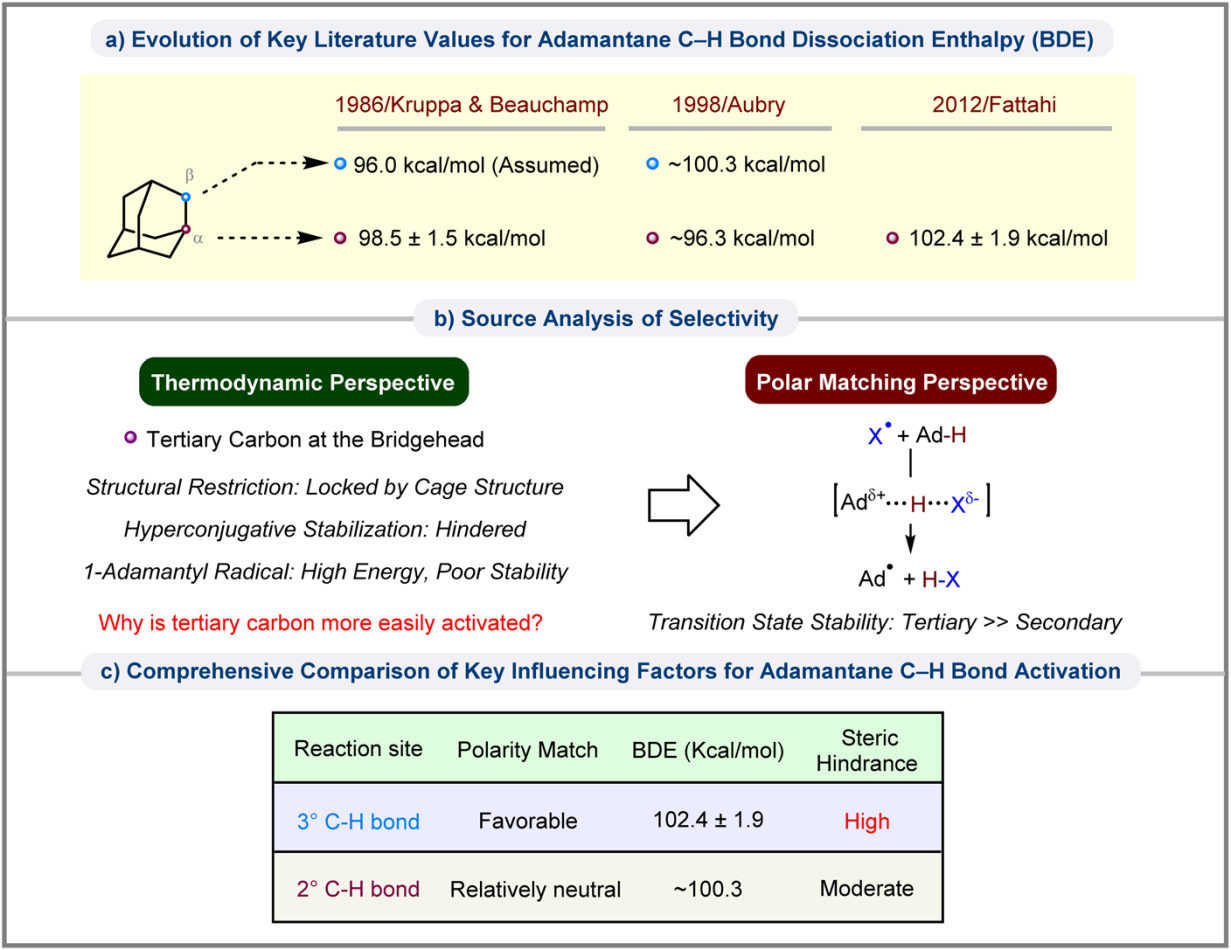
Analysis of the inherent dichotomy in the radical-based C(sp^3^)–H bond activation of adamantane. (a) Evolution of key literature values for adamantane C–H bond dissociation enthalpy (BDE). (b) Source analysis of selectivity from thermodynamic and polar matching perspectives. (c) Comprehensive comparison of key influencing factors for adamantane C–H bond activation.

To resolve this paradox, it is essential to first examine the intricate physicochemical properties of adamantane, as illustrated in Fig. 5. From a thermodynamic standpoint, the origin of its selectivity primarily resides in the C–H bond BDEs, the values of which have been the subject of extensive debate over several decades. An early and influential study by Beauchamp in 1986 reported a bridgehead (tertiary, α) C–H BDE of 98.5 ± 1.5 kcal mol^−1^, derived from thermodynamic cycles and assumptions about reference hydrocarbons commonly accepted at the time.^42^ In contrast, appearance energy measurements conducted by Aubry, Holmes, and Walton in 1998 indicated a higher BDE for the secondary (β) C–H bond—approximately 100.3 kcal mol^−1^—while assigning a lower value of ∼96.3 kcal mol^−1^ to the tertiary (α) C–H bond, thereby challenging the conventional understanding of bond stability.^43^ This discrepancy was further clarified in 2012 through a collaborative effort by the Fattahi and Kass groups, who combined gas-phase experimental data with high-level G3 theoretical calculations.^44^ Their results established a definitive bridgehead (tertiary, α) C–H BDE of 102.4 ± 1.9 kcal mol^−1^, notably higher than that of the secondary (β) C–H bonds (∼100.3 kcal mol^−1^). This elevated BDE at the tertiary position arises from adamantane's rigid cage architecture, which constrains the bridgehead carbon to retain a high-energy pyramidal geometry following radical formation, thus impeding relaxation to a planar sp^2^ configuration and limiting hyperconjugative stabilization.

In contrast to this thermodynamic destabilization, a kinetic polar matching effect plays a critical role.^45^ The electron-donating nature of alkyl substituents renders the tertiary C–H site more electron-rich. When reacting with an electrophilic HAT reagent (X˙), the transition state features charge separation that stabilizes the interaction at the tertiary carbon center. Consequently, adamantane's regioselectivity reflects a delicate balance between opposing factors: tertiary C–H bonds benefit from favorable polar interactions but are hindered by high BDE (102.4 kcal mol^−1^) and significant steric congestion; secondary C–H bonds, while possessing lower BDE (∼100.3 kcal mol^−1^) and moderate steric hindrance, lack advantageous electronic polarization. This interplay leads to the divergent selectivity patterns observed across various catalytic systems.

**Fig. 6:**
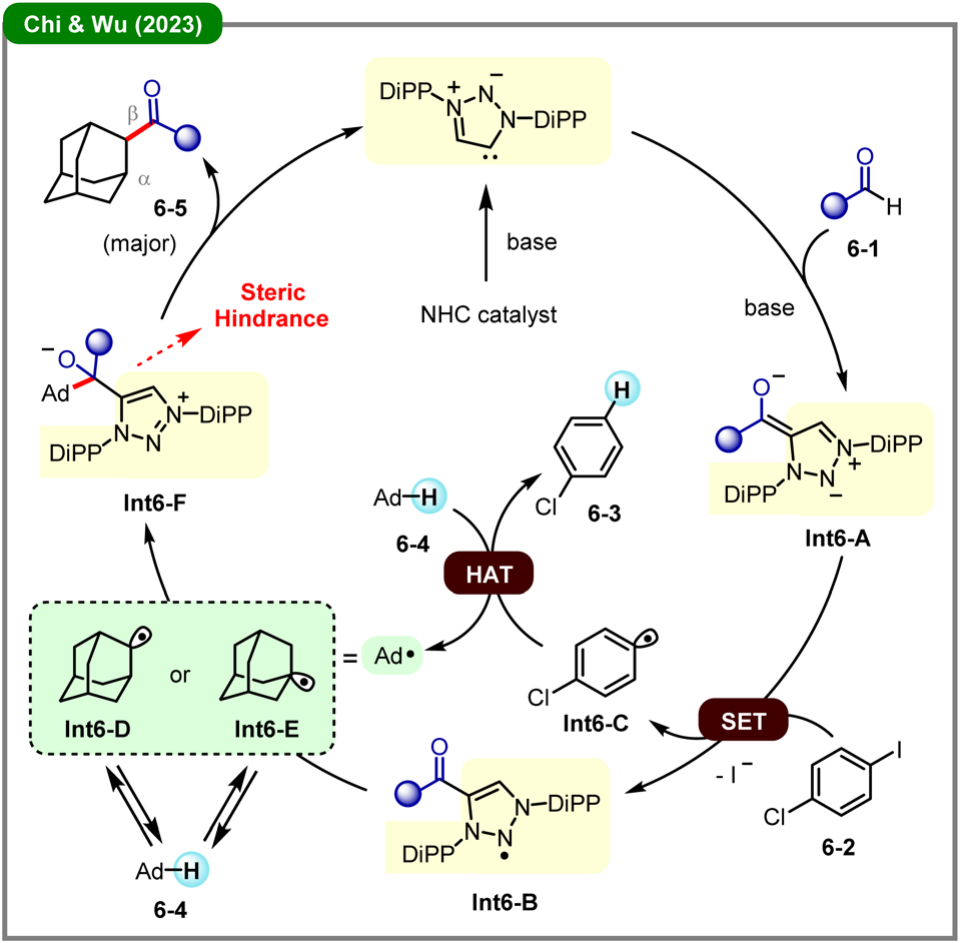
Proposed kinetic trapping mechanism as the origin of selectivity in Chi and Wu's work.

Against this multifaceted backdrop, the origin of selectivity in Chi and Wu's NHC catalytic system^40^ can be reassessed within the context of our framework (Fig. 6). Although the initial HAT between the alkane substrate (6-4) and the *in situ*-generated aryl radical (Int6-C) may exhibit limited selectivity, the subsequent radical–radical coupling step governs the overall regiochemical outcome. The resulting adamantyl radicals (Int6-D and Int6-E) must undergo capture by a sterically demanding acyl-azolium intermediate (Int6-B). At this stage, the tertiary radical (Int6-E), being more sterically encumbered, faces greater kinetic barriers compared to the secondary radical (Int6-D). As a result, the reaction pathway is kinetically favored toward the formation of the β-selective product 6-5 as the major regioisomer.

The so-called “adamantane paradox” therefore highlights the conflict between electronically favored α-site activation and structural limitations arising from molecular rigidity. In our proposed model, β-selectivity predominates when significant steric hindrance—derived from either the “Front-end” abstractor or the “Back-end” receptor—outcompetes the intrinsic electronic preference. This principle, which states that regioselectivity is governed by the region of greatest steric congestion, lays a solid basis for the comparative analysis of photocatalytic systems in the subsequent section.

### 2.4 Case study III: dissecting “front-end” *vs.* “back-end” control in photocatalytic C(sp^3^)–H alkynylation

Following discussion on the “adamantane paradox”, photocatalytic C(sp^3^)–H alkynylation reactions serve as another excellent parallel model for dissecting the two core modes within our kinetic framework. By comparing two mechanistically distinct yet synthetically analogous alkynylation systems—one initiated by the excited state of tetrabutylammonium decatungstate (TBADT*)^46^ and the other by chlorine radical (Cl˙)^47^—we can elucidate how reaction selectivity is regulated either during the initial “Front-end Attack” step or the subsequent “Back-end Capture” step.

**Fig. 7:**
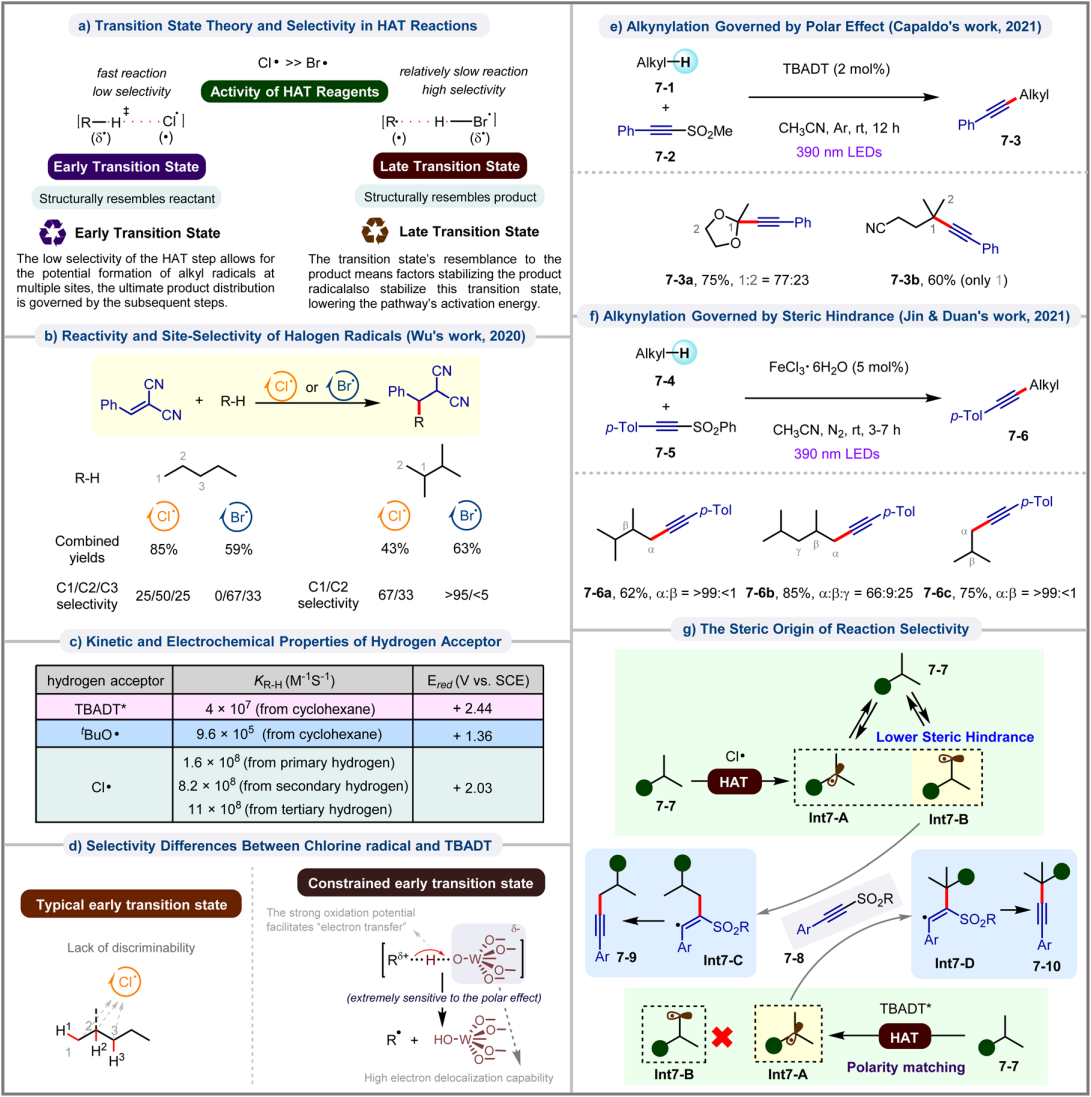
Dissecting “Front-end” and “Back-end” kinetic control in photocatalytic C(sp^3^)–H alkynylation. (a) Transition state theory and selectivity in HAT reactions. (b) Reactivity and site-selectivity of halogen radicals. (c) Kinetic and electrochemical properties of hydrogen acceptors. (d) Selectivity differences between chlorine radical and photoexcited TBADT*. (e) Alkynylation governed by polar effect. (f) Alkynylation governed by steric hindrance. (g) The steric origin of reaction selectivity.

Understanding selectivity requires revisiting the classical Hammond Postulate,^48^ which can be exemplified by hydrogen abstraction processes by halogen radicals (Fig. 7a). Bromine radical (Br˙) reacts slowly and endothermically, with a transition state close to the products (*i.e.*, “late transition state”). Its selectivity thus reflects radical stability, favoring tertiary > secondary > primary C(sp^3^)–H bonds. In contrast, chlorine radical (Cl˙) reacts rapidly and exothermically, with a transition state resembling reactants (*i.e.*, “early transition state”). Here, selectivity is low because the transition state energy is insensitive to final radical stability.^49^ The experimental data from Wu's group^50^ confirm this (Fig. 7b): for 2,3-dimethylbutane, Br˙ attacks the tertiary site selectively (>95 : 5), while Cl˙ shows much lower preference (67 : 33). While the Hammond Postulate explains these trends, it fails to account for differences between alkynylation reactions mediated by Cl˙ and TBADT*. Both highly reactive species should exhibit early transition states and low selectivity, yet experiments show otherwise. This discrepancy demands deeper analysis of their kinetic^51,52^ and electrochemical^53–55^ properties.

The core distinction can be elucidated by comparing Cl˙ and photoexcited TBADT* (Fig. 7c). Cl˙ undergoes hydrogen abstraction at extraordinarily high rates, with rate constants *k*_R–H_ up to 10^8^ M^−1^ s^−1^. Notably, it abstracts inert primary hydrogens faster than common reagents. Its ultrahigh reactivity leads to a classic early transition state (Fig. 7d, left), in which C–H bond scission and charge separation are barely developed. As such, the process is largely unaffected by steric and electronic disparities, ultimately affording non-selective H-abstraction. Conversely, photoexcited TBADT* behaves differently. Despite slightly smaller rate constants (*e.g.*, *k*_R–H_ ≈ 4 × 10^7^ M^−1^ s^−1^, cyclohexane as the substrate), it has a considerably higher reduction potential (*E*_red_ = +2.44 V *vs.* SCE) relative to Cl˙ (+2.03 V). The strong electron-transfer nature of this species yields a constrained early transition state (Fig. 7d, right) with pronounced charge separation ([R^δ+^⋯H⋯(W)^*δ*−^]‡). This carbocation-like transition state leads to partial positive charge localization on the substrate carbon. Under this scenario, selectivity is controlled not by radical stability, but by the polar effects that govern positive-charge stabilization at distinct molecular positions.

The TBADT-photocatalyzed alkynylation (Fig. 7e)^46^ thus can be classified as a “Front-end Attack Control” process, where selectivity is governed by electronic effects during the HAT step. The transition state's strong charge-transfer character enables the catalyst to act as an “electronic filter”, preferentially activating electron-rich α-oxy or tertiary sites that stabilize partial positive charge, even over more sterically accessible positions (*e.g.*, 7-3a and 7-3b). Therefore, selectivity is set irreversibly at the initial bond cleavage, excluding electron-deficient pathways. In contrast, the iron-photocatalyzed alkynylation (Fig. 7f)^47^ follows the “Back-end Capture Control”. After non-selective alkyl radical generation by Cl˙, bulky alkynyl sulfones enforce selectivity during capture. Capturing a large tertiary radical incurs a high steric penalty—similar to cavity formation energy (*v*V_x_) in LSER models—creating a kinetic barrier much higher than for primary radicals (Fig. 7g). As a result, the Curtin–Hammett principle directs the radical equilibrium toward terminal products. This is supported by high terminal selectivity (*α* : *β* > 99 : 1) in sterically differentiated substrates (*e.g.*, 7-6a and 7-6c). These photocatalytic reactions offer a clear comparison of two kinetic control mechanisms: “Front-end Attack Control” uses the hydrogen abstractor's intrinsic electronic or steric bias to filter pathways at cycle entry; “Back-end Capture Control” enforces selectivity at cycle exit *via* a steric trap, which requires a non-selective HAT step (*e.g.*, using highly reactive Cl˙) to generate a “radical pool” for downstream discrimination.

### 2.5 Case study IV: mechanistic validation of “back-end capture” through the “radical sampling” paradigm

**Fig. 8:**
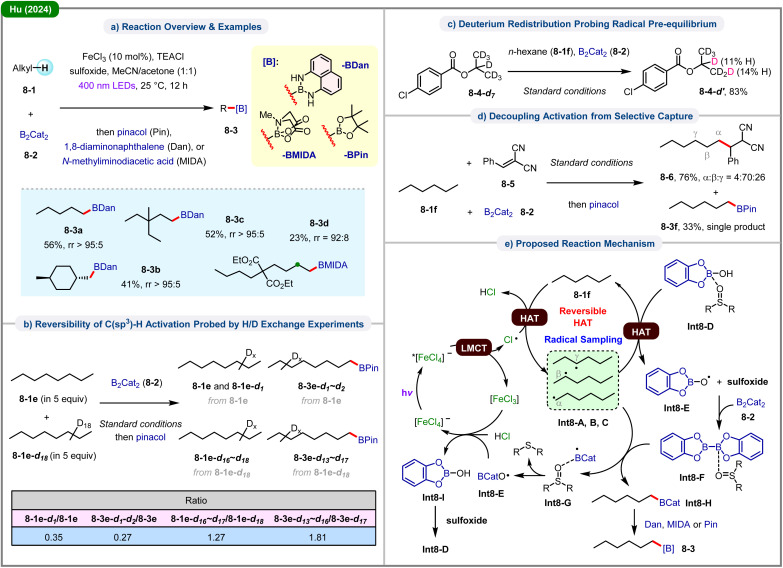
Terminal C(sp^3^)–H borylation *via* “radical sampling” paradigm. (a) Reaction overview & examples. (b) Reversibility of C(sp^3^)–H activation probed by H/D exchange experiments. (c) Deuterium redistribution probing radical pre-equilibrium. (d) Decoupling activation from selective capture. (e) Proposed reaction mechanism.

Although the “Back-end Capture” mechanism is theoretically sound, its foundational assumptions—namely, the reversibility of non-selective HAT and the ensuing kinetic discrimination—have remained experimentally unverified until recently. The “radical sampling” strategy, introduced by Hu and colleagues in 2024 (Fig. 8),^56^ furnishes conclusive mechanistic evidence supporting this model. Specifically, their iron-catalyzed borylation of linear alkanes (8-1) with bis(catecholato)diboron (B_2_Cat_2_, 8-2) demonstrates how high terminal selectivity can be realized despite an initially non-selective radical generation step.

To establish a “sampling-and-filtering” mechanism, the authors conducted a comprehensive mechanistic investigation. H/D exchange experiments confirmed the reversibility of the HAT step, thereby establishing a dynamic equilibrium among alkyl radicals and their parent alkanes (Fig. 8b and c). More critically, decoupling control experiments demonstrated that when the carbon radicals are intercepted by an electron-deficient olefin (8-5, a Giese-type acceptor) instead of the bulky boron species derived from 8-2, a mixture of regioisomers (8-6) was obtained (Fig. 8d). These findings provide unambiguous evidence that selectivity is not encoded the initial C–H cleavage, but is instead imposed exclusively at the downstream (*i.e.*, “Back-end”) radical capture step. As summarized in the integrated catalytic cycle (Fig. 8e), the photoinduced ligand-to-metal charge transfer (LMCT) generated Cl˙ initiates reversible HAT to form a transient alkyl radical pool; selective interception of terminal radicals by the bulky boron–sulfoxide complex (Int8-F) then acts as the decisive kinetic gate, controlling product formation at the cycle's terminus.

This case study demonstrates the broader conceptual value of our framework in rationalizing undirected C(sp^3^)–H functionalization reactions. The consistency between the “radical sampling” paradigm and the “Back-end Capture Control” model reveals a universal governing principle: terminal selectivity arises from strategically located kinetic bottlenecks within the catalytic cycle. Such rate-limiting constraints can occur at either the entry point (“Front-end”) or, as showcased in this system, the exit point (“Back-end”) of the catalytic manifold.

### 2.6 Case study V: the multidimensional role of solvation effects

Although Section 2.1 has briefly acknowledged the influence of solvents, a more systematic investigation into their dual role as kinetic and electronic modulators is essential to advance our mechanistic understanding of terminal C(sp^3^)–H bond selectivity. Properties such as coordination ability, polarity, and hydrogen-bond donor (HBD) strength, collectively dictate solvation effects—either by stabilizing radical intermediates or perturbing substrate electronics—thus fundamentally altering HAT kinetics.^32,34,57^

**Fig. 9:**
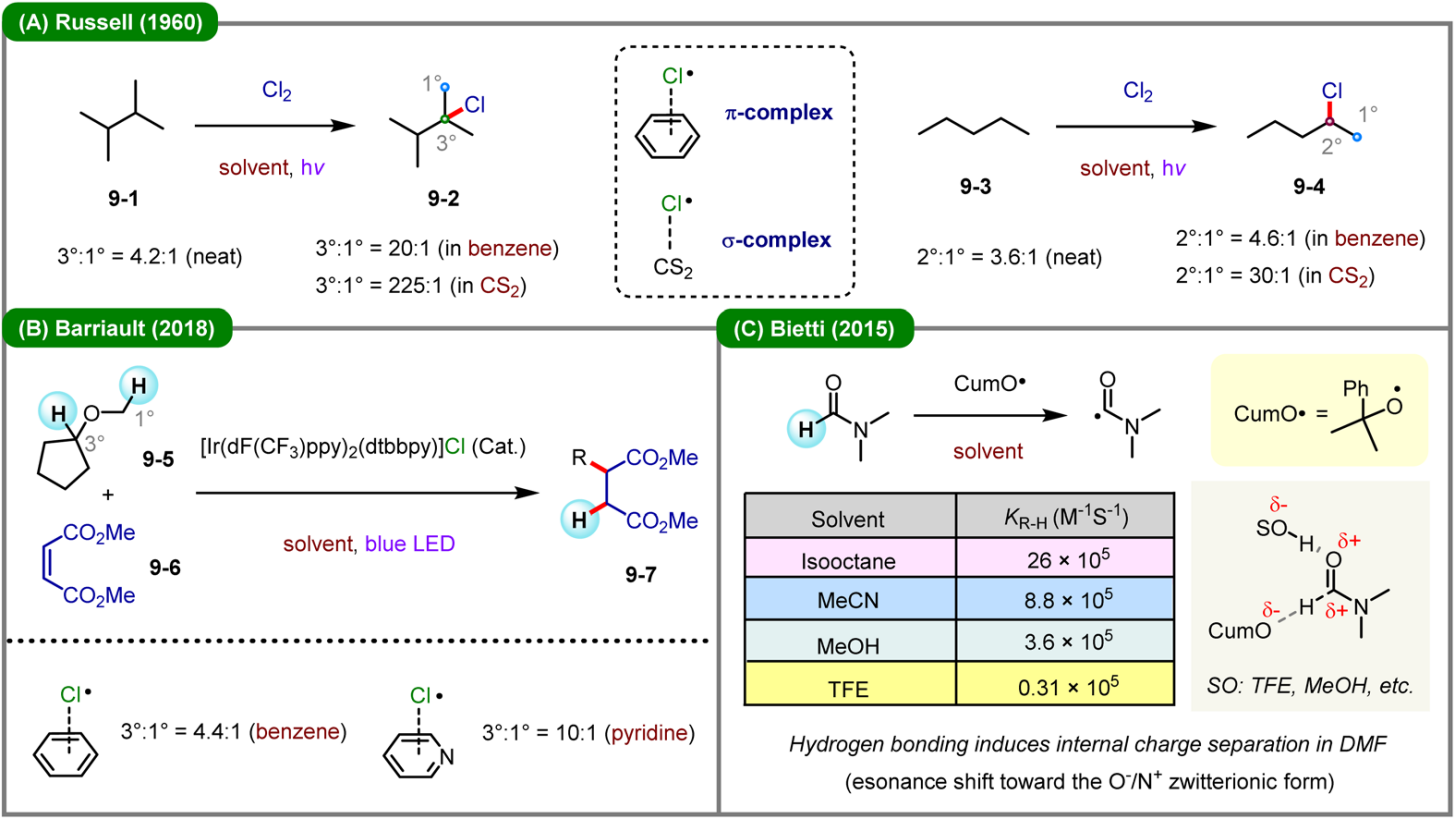
Kinetic and electronic modulation of solvation effects on terminal-selective C(sp^3^)–H functionalization through radical complexation and substrate deactivation. (A) Russell's work; (B) Barriault's work; (C) Bietti's work.

Russell's foundational studies in 1960 illustrate how solvent coordination directly modulates radical reactivity (Fig. 9A).^57^ Chlorine radical, which displays minimal selectivity in the gas phase, undergo pronounced selectivity enhancement in coordinating solvents. For instance, aromatic solvent benzene engages Cl˙ *via* π-complexation, thereby attenuating its electrophilicity and reactivity. Even more striking is the effect observed in carbon disulfide (CS_2_), where Cl˙ forms a solvent-bridged σ-complex. This interaction not only reduces the radical's electrophilic character but also substantially increases its effective steric volume, thereby reducing relative reactivity toward primary C(sp^3^)–H bonds by up to two orders of magnitude. As illustrated in Fig. 9B, Barriault's 2018 photocatalytic chlorine radical-mediated HAT system further verifies that solvent-regulated radical modulation serves as the core origin of selectivity control.^34^ The use of coordinating solvents such as benzene and pyridine effectively attenuates the inherent high reactivity of Cl˙, thereby “taming” the radical species to enable precise C–H bond discrimination based on electronic and steric disparities. This strategy extends the classical solvation effect framework originally proposed by Russell to contemporary catalytic scenarios.

In contrast, Bietti's work (Fig. 9C)^32^ highlights the unique regulatory role of strong HBD solvents, with 2,2,2-trifluoroethanol (TFE) serving as a representative example. For substrates bearing polar functional groups such as amides and esters, TFE forms robust hydrogen bonds with the carbonyl oxygen of these moieties. Such noncovalent interactions strengthen the electron-withdrawing capability of carbonyl groups, amplifying substrate inductive polarization and consequently suppressing HAT reactivity at the adjacent α- and β-C–H sites.

Collectively, these case studies demonstrate that solvents exert multifaceted regulation over HAT selectivity, which can be categorized as the “Front-end Attack Control” model. Three complementary solvent-governed mechanisms account for such selectivity modulation: (1) tuning the steric and electronic properties of abstracting radicals through solvent coordination or complexation;^34,57,58^ (2) enabling site-specific modulation of substrate reactivity *via* noncovalent interactions, including hydrogen bonding and dipole–dipole interactions;^32,59^ and (3) dynamically stabilizing polarized transition states through solvent polarity effects, which facilitates polarity-reversal reaction pathways to override intrinsic BDE-governed reactivity preferences.^59,60^

From a kinetic standpoint, solvation effects act as a dynamic regulatory lever to shift reaction behavior across the “kinetic continuum” (discussed in the following section), thereby steering reaction systems toward either “Front-end” or intermediate kinetic control regimes. Specifically, noncovalent interactions and charge-transfer coordination between solvent molecules and radical abstractors mitigate the intrinsic homolytic reactivity of radical species. This solvent-induced “taming” effect raises the activation barrier of the initial HAT step, converting unselective, highly exothermic radical attack into a structurally discriminating, kinetically governed reaction pathway. In this context, rational solvent selection emerges as an elegant nonstructural strategy for fine-tuning “Front-end” kinetic bottlenecks in selective C(sp^3^)–H functionalization.

### 2.7 Bridging idealized regimes toward a “kinetic continuum”

**Fig. 10:**
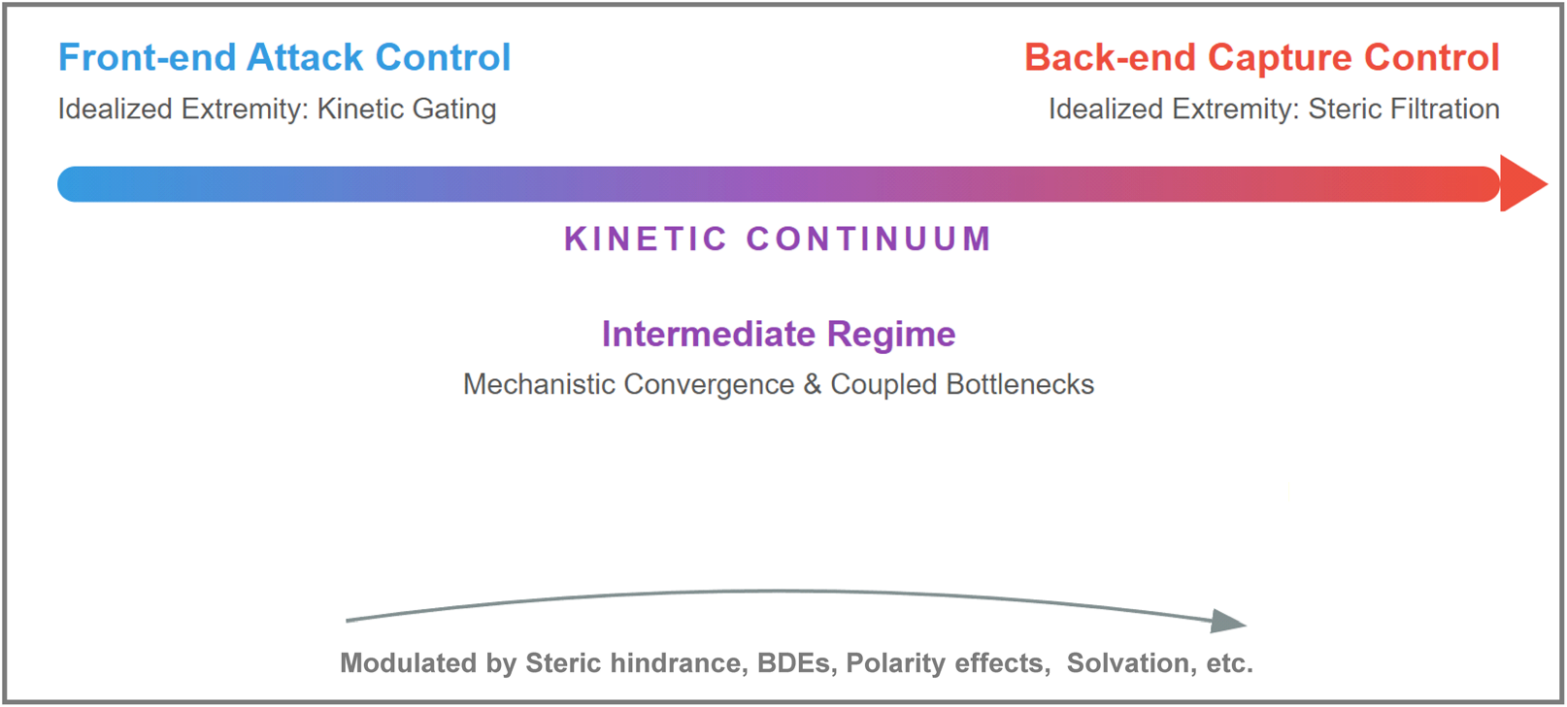
Conceptual representation of the “kinetic continuum” for terminal-selective C(sp^3^)–H functionalization.

The preceding discussions (Sections 2.1–2.6) have addressed the foundational principles governing terminal selectivity in HAT-mediated radical C(sp^3^)–H functionalization. Although our dichotomous classification into “Front-end Attack Control” and “Back-end Capture Control” offers a conceptually unifying framework, it is critical to emphasize that these categories represent idealized limiting cases situated along a continuous “kinetic continuum” (Fig. 10).

In practical catalytic systems, site selectivity is rarely determined by a single well-defined transition state. Instead, most reactions proceed *via* the “intermediate regime”, where the observed terminal selectivity stems from the combined kinetic and thermodynamic effects modulated collectively by steric environment, BDEs, electronic polarity and solvation effects. For example, when the HAT step becomes reversible, polarity matching or thermodynamic radical partitioning can outweigh the steric control imposed by “Front-end” kinetic gating. Treating terminal selectivity as a dynamic position along this kinetic continuum enables researchers to properly interpret “boundary cases” that feature hybrid control mechanisms.

With this continuum framework as the guideline, the rest of this review presents a systematic classification of undirected C(sp^3^)–H functionalization approaches. Section 3 focuses on systems dominated by “Front-end Attack Control”, wherein regioselectivity is predominantly determined at the initial C–H cleavage step *via* physical confinement, catalyst-imposed steric hindrance, or intrinsic thermodynamic bias. Section 4 addresses “Back-end Capture Control”, elucidating how sterically encumbered transition-metal complexes or main-group reagents enforce terminal selectivity following a non-selective, pre-equilibrated HAT step. Finally, Section 5 moves beyond these idealized extremes to examine synergistic multi-step relay catalysis, thermodynamic or polarity-driven overrides, and representative examples of the “intermediate regime”, thereby demonstrating how this continuous kinetic framework operates in more complex catalytic environments.

## 3. “Front-end” control of site selectivity

“Front-end Attack Control” is defined as the scenario where regioselectivity is locked in at the entry of the catalytic cycle, prior to any external trapping event. This mode operates through two distinct yet complementary mechanisms: (1) kinetic filtration: where a restricted steric environment (*e.g.*, enzyme pockets or bulky ligands) creates a “trajectory cone” that physically excludes the approach of internal C–H bonds; (2) thermodynamic selection/equilibration: where sterically congested intermediates, once formed, undergo rapid isomerization to the thermodynamically more stable terminal position driven by relief of steric strain. Guided by this logic, this chapter demonstrates how selectivity is enforced either by creating a steric bottleneck for entry or by driving an equilibrium toward the terminal intermediate.

### 3.1 Control based on physical confinement and molecular cavities

The discussion first explores how physical confinement creates molecular “reaction tunnels” for precise terminal recognition, through the application enzyme active sites or zeolite pores.

**Fig. 11:**
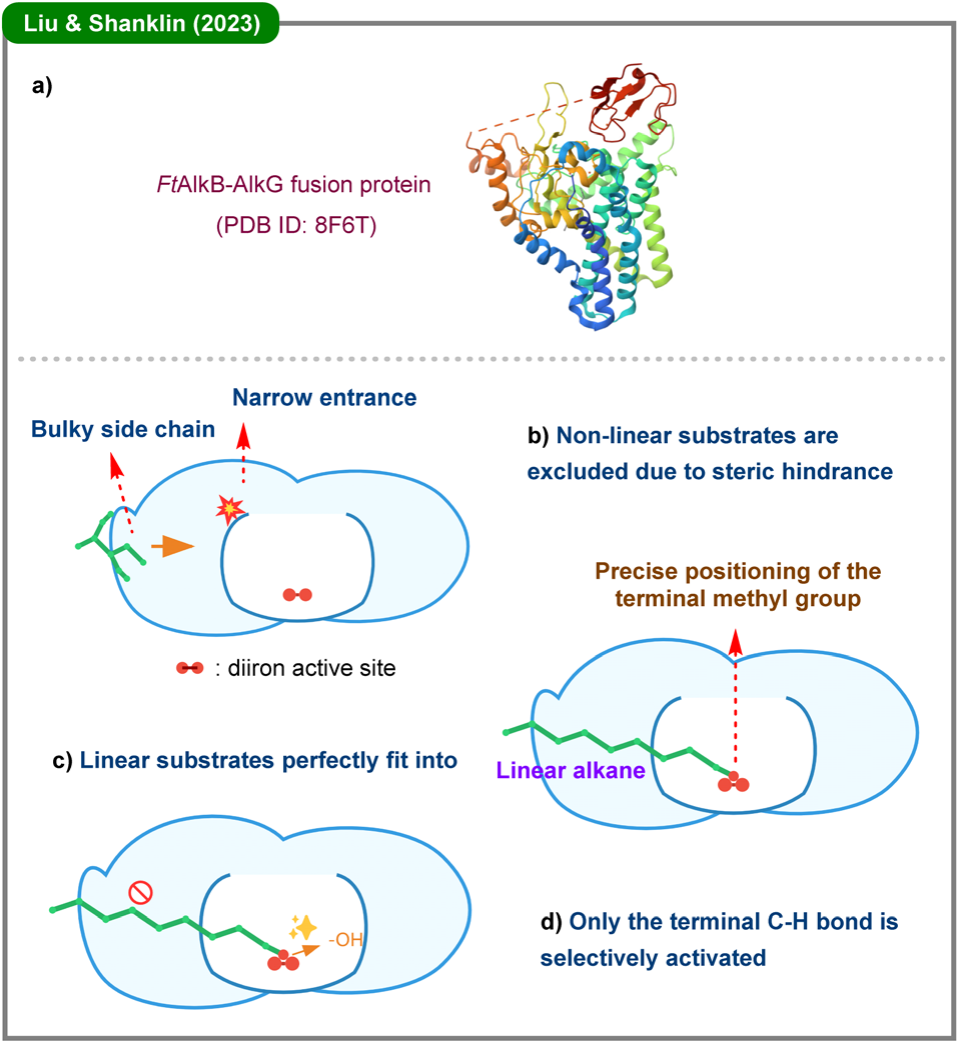
Enzyme catalysis through the “shape-selectivity” of AlkB active pocket for terminal C–H hydroxylation. (a) Structure of *Ft*AlkB–AlkG fusion protein. (b) Exclusion of non-linear substrates due to steric hindrance. (c) Linear substrates perfectly fitting into the pocket. (d) Selective activation of the terminal C–H bond.

Enzymes, nature's highly evolved catalysts, achieve exceptional efficiency and selectivity.^61^ For instance, cytochrome P450 enzymes like CYP153A6 convert medium-chain alkanes (C6–C11) to terminal alcohols with over 95% selectivity,^62,63^ while alkane monooxygenase (AlkB) enzymes show distinct chain-length preferences.^64^ In 2023, the Liu and Shanklin groups used cryo-electron microscopy to resolve the high-resolution structure of a natural AlkB–AlkG fusion protein from *Fontimonas thermophila* (*Ft*AlkB), providing key insights into enzymatic terminal selectivity (Fig. 11).^65^ The high-resolution structure reveals a long, narrow hydrophobic tunnel that acts as a selective substrate channel (Fig. 11a). This confined space serves as a gatekeeper: bulky or branched alkanes are blocked at the entrance, while linear alkanes fit smoothly (Fig. 11b and c). Lining hydrophobic residues guide the substrate so that its terminal methyl group faces the diiron active site, ensuring only the terminal C(sp^3^)–H bond is activated (Fig. 11d). Internal C–H bonds are either too far or sterically shielded, making them inaccessible. This “shape-selectivity” mechanism exemplifies a “Front-end Attack Control” strategy, where physical confinement determines the reaction site. Although this principle inspires biomimetic catalyst design, it is currently limited to alkane functionalization by enzymes.

**Fig. 12:**
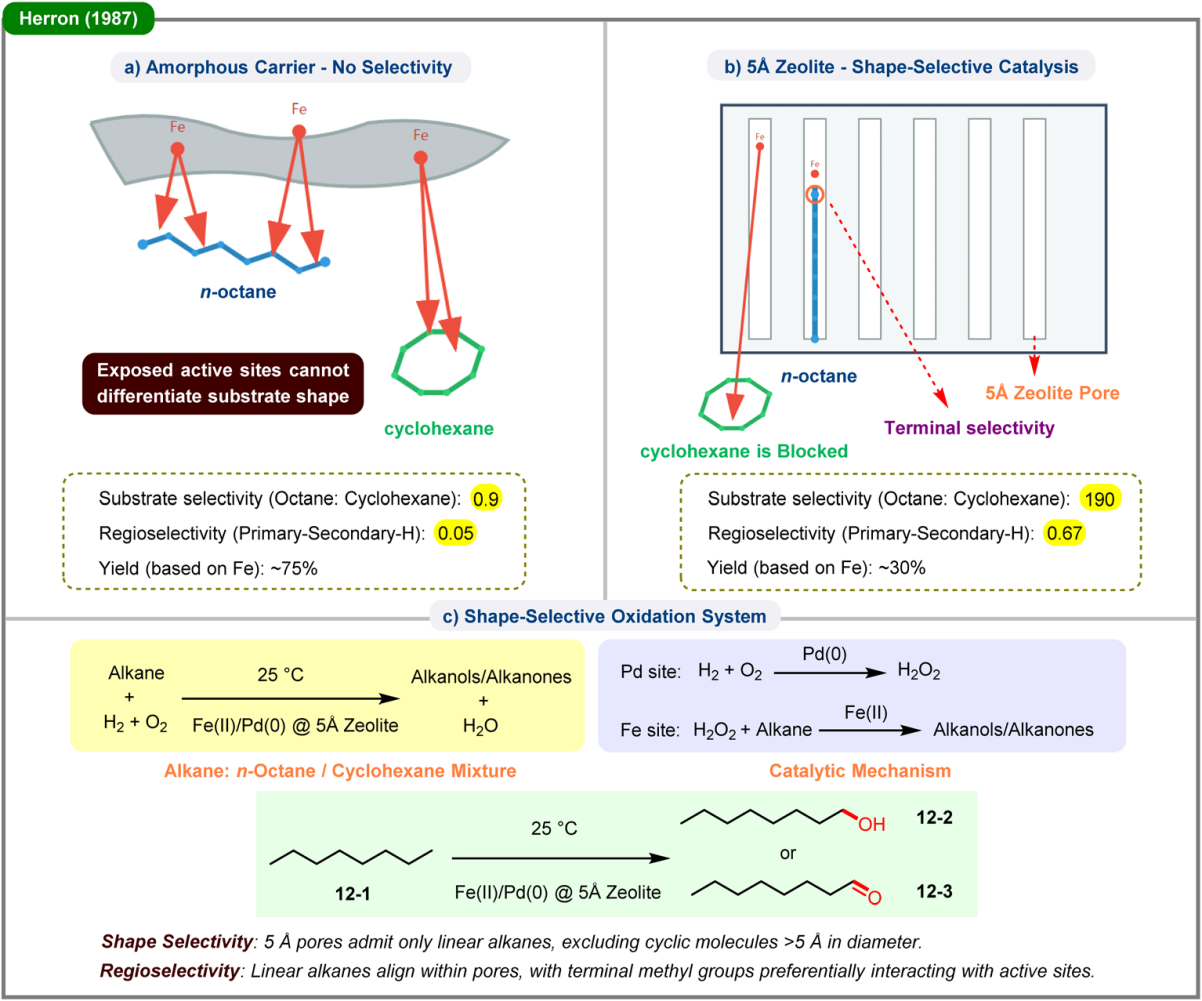
Zeolite catalysis demonstrating shape-selectivity *via* rigid micropores. (a) Amorphous carrier showing no selectivity. (b) 5 Å zeolite demonstrating shape-selective catalysis. (c) Scheme and catalytic mechanism of the shape-selective oxidation system.

Using the microporous structure of zeolites to mimic enzymatic selectivity^66^ represents another classic “Front-end Attack Control” approach. Herron's 1987 work on a “completely inorganic mimic of alkane ω-hydroxylases” pioneered this strategy (Fig. 12).^9^ To test their concept, the authors first used an amorphous silico–aluminate support with exposed active sites (Fig. 12a). This non-shape-selective catalyst showed almost no preference between *n*-octane and cyclohexane (oxidation ratio: 0.9) and poor regioselectivity (primary-to-secondary C–H ratio: 0.05), confirming that the active sites lack intrinsic selectivity. In contrast, a 5 Å zeolite catalyst showed dramatic improvement (Fig. 12b). Its rigid pores (∼5 Å) allow linear alkanes like *n*-octane to enter while blocking bulkier molecules like cyclohexane. This physical gate enables high substrate selectivity, increasing the octane/cyclohexane oxidation ratio to 190. Inside the pore, the alkane adopts an extended conformation, positioning the terminal methyl group closest to the iron active sites. This geometric constraint achieves elevated terminal (ω-site) selectivity, with a primary-to-secondary oxidation ratio of 0.67. The overall system reflects elegant design, where Pd(0) and Fe(ii) sites are embedded in the zeolite framework (Fig. 12c). Under H_2_ and O_2_, palladium generates hydrogen peroxide *in situ*, which drives Fenton-type oxidation at the iron centers. This study proves that artificial confinement can control substrate orientation, enabling precise site-selectivity through “Front-end” reaction engineering.

### 3.2 σ-Bond activation based on catalyst steric hindrance

In transition-metal-catalyzed systems, a core activation strategy involves the direct σ-bond interaction between a C–H bond and the metal center. In such reactions, selectivity is primarily governed by the steric hindrance of the catalyst's ligands, which creates a kinetic bias favoring reaction with the least encumbered terminal C(sp^3^)–H bond.^67–69^ This principle is realized through several distinct mechanism patterns depending on the specific nature of the C–H activation step.

#### 3.2.1 Steric effects in the σ-bond metathesis pathway

**Fig. 13:**
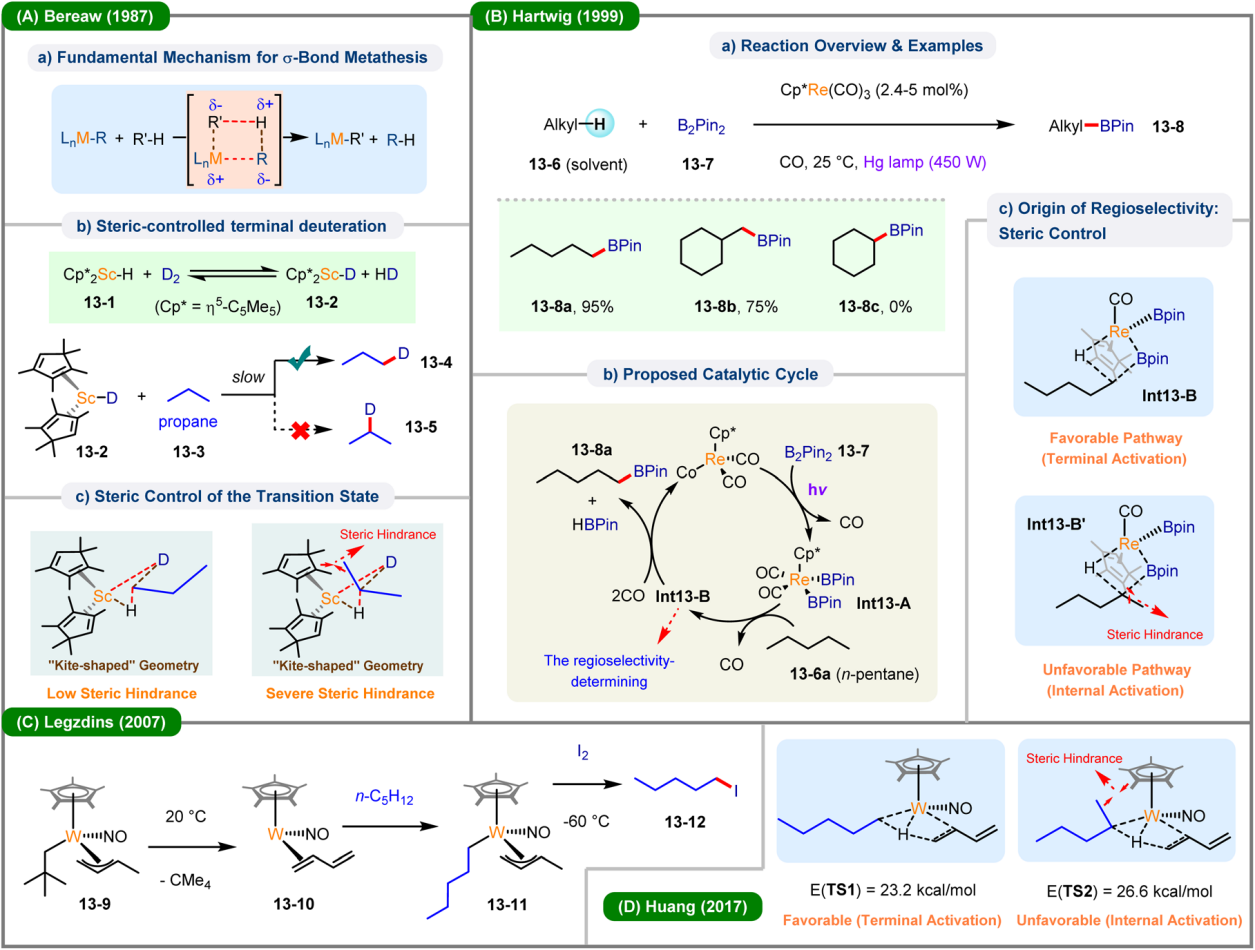
Steric effects in the σ-bond metathesis pathway. (A) Sc-catalyzed terminal C–H deuteration: (a) fundamental mechanism, (b) steric-controlled terminal deuteration, (c) steric control of the transition state. (B) Re-catalyzed terminal C–H borylation: (a) reaction overview & examples, (b) proposed catalytic cycle, (c) origin of regioselectivity *via* steric control. (C) Terminal C–H activation and iodination of *n*-pentane mediated by a tungsten nitrosyl complex. (D) Density functional theory calculations on the *n*-pentane terminal iodination.

The σ-bond metathesis pathway is a concerted mechanism common in electron-deficient early transition-metal complexes, particularly those with d^0^ configurations. Bercaw's work on permethylscandocene 
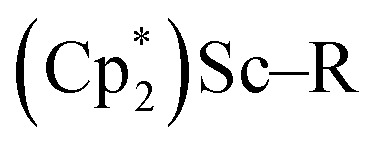
 complexes illustrates how steric bulk can enforce terminal selectivity within this framework (Fig. 13A).^68^ The reaction proceeds through a four-center, kite-shaped transition state where the substrate's C–H bond interacts directly with the M–R bond, enabling R and H exchange. The d^0^ scandium center is shielded by two bulky pentamethylcyclopentadienyl (Cp*) ligands. In H/D exchange with propane (13-3), the catalyst reacts exclusively with terminal methyl C–H bonds, while internal methylene bonds remain inert. This selectivity arises from steric repulsion: when the terminal C–H bond approaches, the alkyl chain orients away from the Cp* groups, resulting in a low-energy transition state. In contrast, activation of an internal secondary C–H bond forces severe clash between a substrate methyl group and a Cp* ligand, greatly increasing activation energy and shutting down this pathway.

The photochemical alkane borylation catalyzed by rhenium, developed by Hartwig's group in 1999, constitutes another classic case of terminal-selective C(sp^3^)–H functionalization (Fig. 13B).^70^ Under irradiation and a CO atmosphere, the catalytic system based on [Cp*Re(CO)_3_] converts alkanes and bis(pinacolato)diboron (B_2_Pin_2_, 13-7) to the corresponding terminal alkylboronate esters (13-8). The functionalization of *n*-pentane affords 13-8a in 95% yield, and no internal isomeric products can be detected. Cyclohexane, possessing solely secondary C–H bonds, exhibits no reactivity (13-8c), demonstrating the exclusive selectivity toward terminal positions. Despite multiple proposed mechanisms such as σ-bond metathesis and oxidative addition, the regioselectivity is governed by steric effects during C–H activation, following the “Front-end Attack Control” model. Transition state analyses reveal that the selectivity-determining step involves the reaction of the bulky rhenium bis-boryl intermediate with alkanes (Int13-B).

Legzdins' study on a tungsten nitrosyl complex further demonstrates steric control in σ-bond metathesis (Fig. 13C).^71^ The designed complex Cp*W(NO)(η^3^-CH_2_CHCHMe)(CH_2_CMe_3_) (13-9) activates the terminal C–H bond of *n*-pentane at room temperature, releasing neopentane and forming a stable *n*-pentyl complex (13-11). Treatment of this compound with I_2_ at −60 °C yields 1-iodopentane (13-12), completing the conversion of inert alkane to terminal functionalized product. Isotopic labeling experiments confirmed exclusive terminal C–H activation, ruling out internal activation followed by rearrangement. In 2017, density functional theory (DFT) calculations by Huang and coworkers confirmed the mechanism as σ-bond metathesis (Fig. 13D).^72^ The calculated activation energy for terminal C–H cleavage (23.2 kcal mol^−1^) is 3.4 kcal mol^−1^ lower than for internal activation (26.6 kcal mol^−1^), due to steric clash between the substrate chain and the Cp* ligand in the internal pathway, which ensures terminal selectivity. Together, experimental and theoretical results provide strong evidence that steric effects in the transition state enable high terminal selectivity in σ-bond metathesis.

#### 3.2.2 Steric effects in the oxidative addition/reductive elimination pathway

In transition-metal catalysis, achieving terminal selectivity *via* oxidative addition (OA)/reductive elimination (RE) sequences is central to the “Front-end Attack Control” strategy. Regioselectivity is determined in the initial C–H activation step, where the catalyst's steric environment hinders reactions at internal C–H bonds, favoring oxidative addition at terminal methyl groups.

**Fig. 14:**
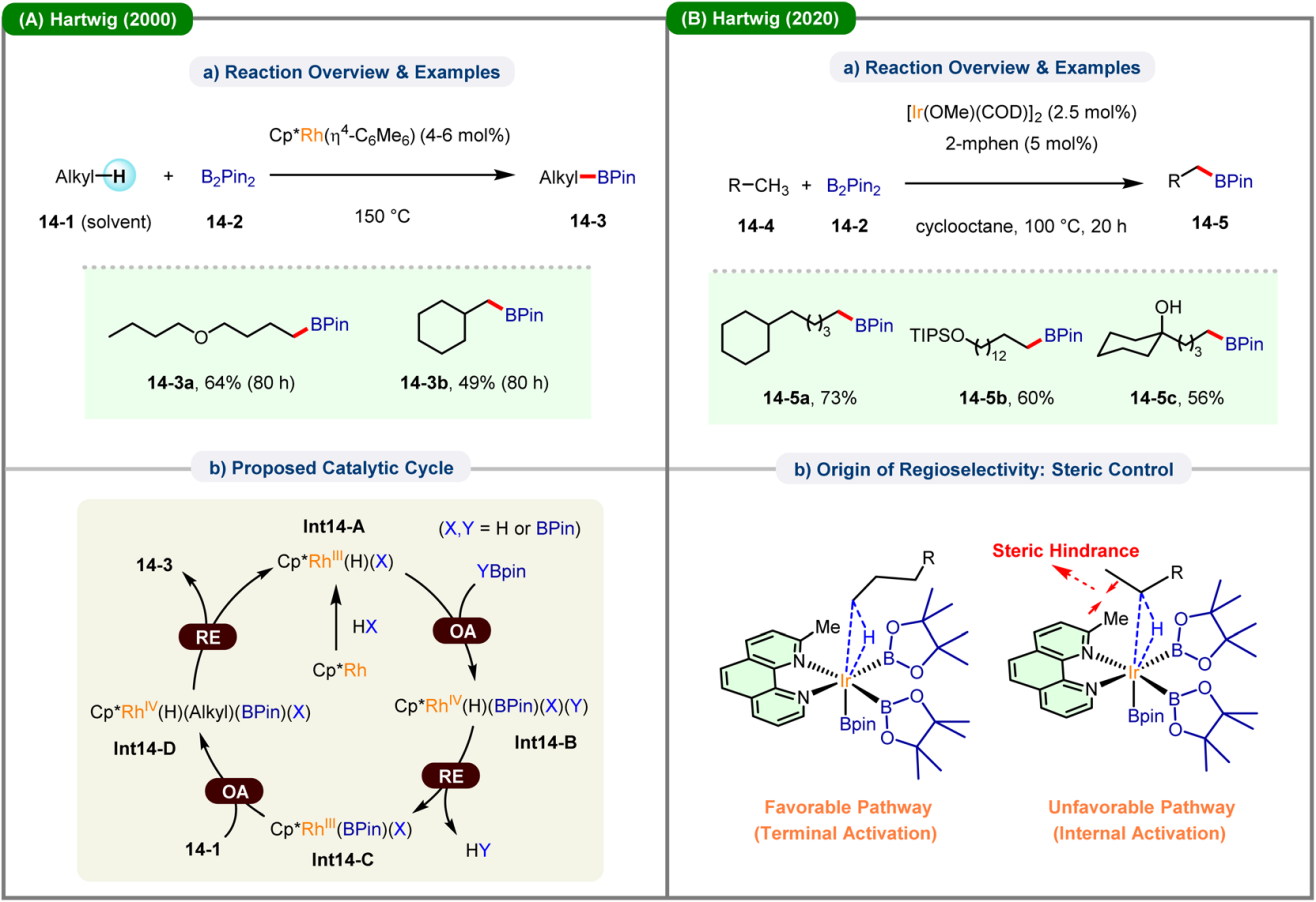
Mechanistic origins of terminal selectivity in Hartwig's rhodium- and iridium-catalyzed alkane borylation. (A) Rh-catalyzed system: (a) reaction overview & examples, (b) proposed catalytic cycle. (B) Ir-catalyzed system: (a) reaction overview & examples, (b) origin of regioselectivity: steric control.

The pioneering rhodium-catalyzed alkane borylation reported by Hartwig's group in 2000 exemplifies this principle (Fig. 14A).^73^ The Cp*Rh(η^4^-C_6_Me_6_) complex selectively functionalizes linear alkanes (14-1) with B_2_Pin_2_ (14-2), yielding exclusively terminal alkylboronate esters (14-3). The authors attributed this to a “steric preference for forming linear metal-alkyl complexes” during C–H activation, which represents a classic case of “Front-end” steric control. A decade later, Hall, Hartwig, and colleagues provided a more refined understanding of the origin of this selectivity through combined detailed H/D exchange experiments and DFT calculations, confirming that the C–H bond cleavage is reversible.^74^ This work demonstrated that primary C–H bond exchange rates are significantly faster than secondary C–H bonds. Notably, with cycloalkanes like cyclohexane, H/D exchange also occurred but no borylation products formed, proving that secondary C–H bonds can be activated, while a high-energy barrier in reductive elimination step blocks product formation. This results in exclusive terminal selectivity at the “Front-end” of the catalytic cycle, driven by kinetic amplification.

In contrast to Rh-catalyzed systems, where terminal selectivity emerges from kinetic amplification in a reversible C–H activation process, iridium catalysts follow a distinct mechanism. Here, oxidative addition is typically irreversible and determines regioselectivity, making steric effects in the catalyst the sole control factor—a clear case of “Front-end” control. Early iridium systems had low activity and required neat substrate conditions, but advances in ligand design have enabled efficient catalysis in practical, solvent-based setups. A major breakthrough came in 2020 from the Hartwig group using a 2-methylphenanthroline (2-mphen) ligand (Fig. 14B).^75^ This ligand greatly accelerated the reaction, allowing undirected borylation of limiting reagent alkanes (14-4) in inert solvents like cyclooctane. High terminal selectivity was traced directly to the C–H activation step. A large kinetic isotope effect (KIE = 3.4 ± 0.2) for *n*-octane confirmed that C–H cleavage is irreversible and rate-determining. The ligand's steric bulk favors attack at terminal methyl groups while blocking access to more hindered internal C–H bonds. In the same year, the Schley group developed a powerful system based on a new 2,2′-dipyridylarylmethane ligand scaffold, enabling efficient borylation of alkanes in cyclohexane with minimal substrate excess.^76^ Although detailed mechanistic studies were limited, the consistent preference for primary C–H bonds points to a similar steric control model. The authors suggest the ligand may adopt a facial κ^3^-binding mode after cyclometalation, creating a crowded environment akin to Cp*-based catalysts known for terminal selectivity.

#### 3.2.3 Steric effects in metal carbene insertion reactions

**Fig. 15:**
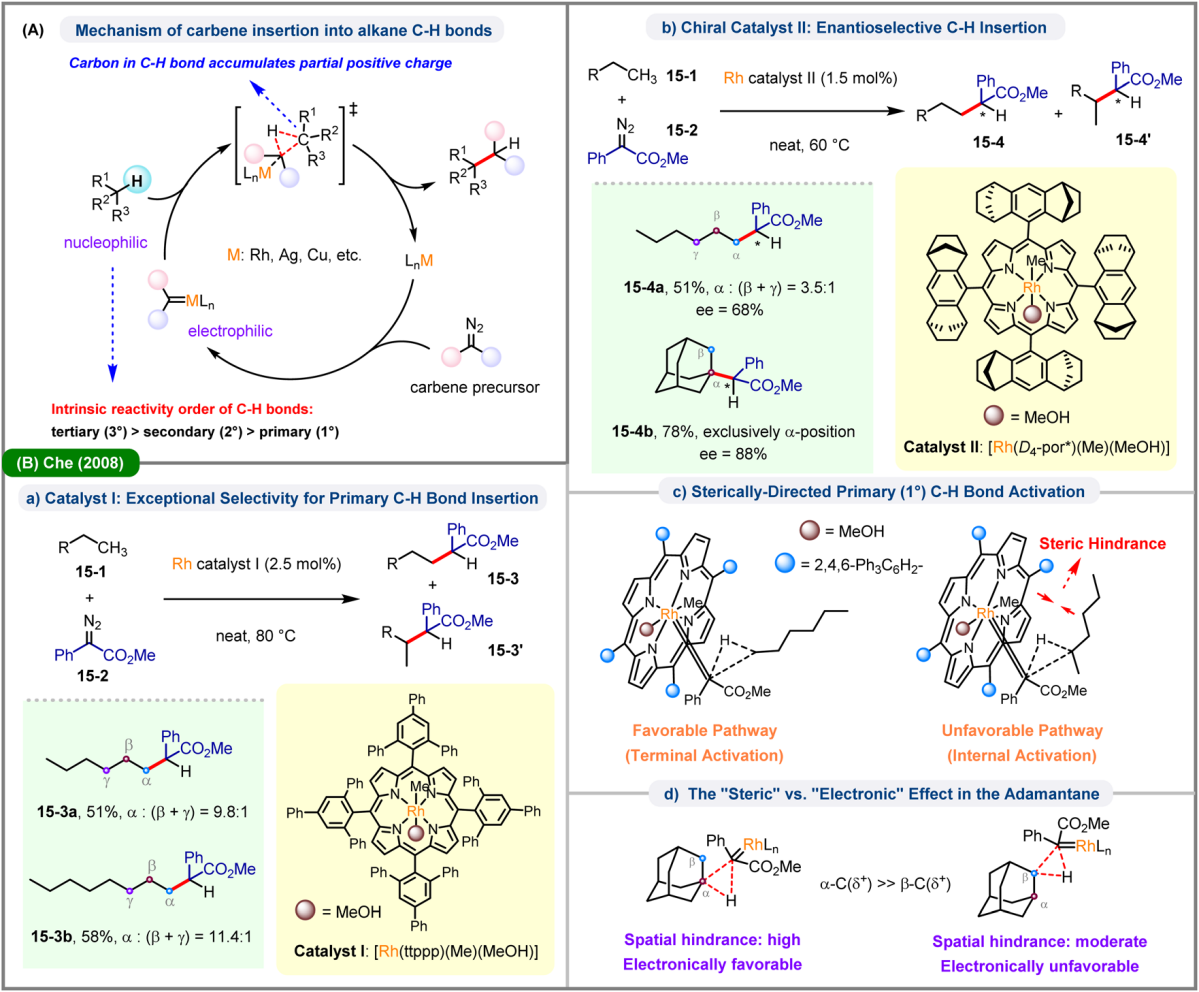
Carbene C(sp^3^)–H insertion *via* sterically confined catalysts. (A) A generally accepted mechanism. (B) Rh-catalyzed carbenoid insertion into primary C–H bonds: (a) Catalyst I with exceptional selectivity; (b) Chiral catalyst II for enantioselective insertion; (c) sterically-directed primary (1°) C–H activation; (d) the “steric” *vs.* “electronic” effect in the adamantane.

In metal carbene-mediated C(sp^3^)–H functionalization reactions, achieving terminal selectivity is challenging due to the inherent electronic preference for more substituted C(sp^3^)–H bonds.^77^ As shown in Fig. 15A, the generally accepted mechanism proceeds *via* electrophilic attack by the metal carbene, forming transition states with “carbocation-like” character. This determines that reactions preferentially occur at tertiary carbon (3°) sites that can better stabilize positive charge, with the inherent reactivity order being: tertiary > secondary > primary.^78,79^ To overcome this bias, the “Front-end Attack Control” strategy has emerged, with its core principle being the utilization of steric hindrance effects from catalysts.

Early studies noted that increased steric bulk, such as at the *ortho*-positions of porphyrin ligands, could modestly enhance primary C–H reactivity.^80,81^ Even with bulky ligands like 9-triptycenecarboxylate, however, selectivity was only modestly improved, still favoring secondary sites. A breakthrough came from Che's group in 2008 (Figure 15B),^10^ who developed a sterically hindered Rh Catalyst I, [Rh(ttppp)(Me)(MeOH)], that reversed the natural reactivity, achieving a high primary/secondary selectivity of 11.4 : 1 in linear alkanes (15-3b). In contrast, the less hindered Catalyst II, [Rh(D_4_-por*)(Me)(MeOH)], showed lower terminal selectivity (15-4a). The spatial model in Fig. 15B-c illustrates how the bulky ttppp ligand creates a confined “pocket” that favors access by terminal methyl groups while blocking internal methylene groups due to steric repulsion. Taking the functionalization of adamantane (15-4b) as an example (Fig. 15B-d), the strategy clearly demonstrates the balance between sterically accessible sites (*i.e.*, β-positions) and electronically favored ones (*i.e.*, α-positions).

**Fig. 16:**
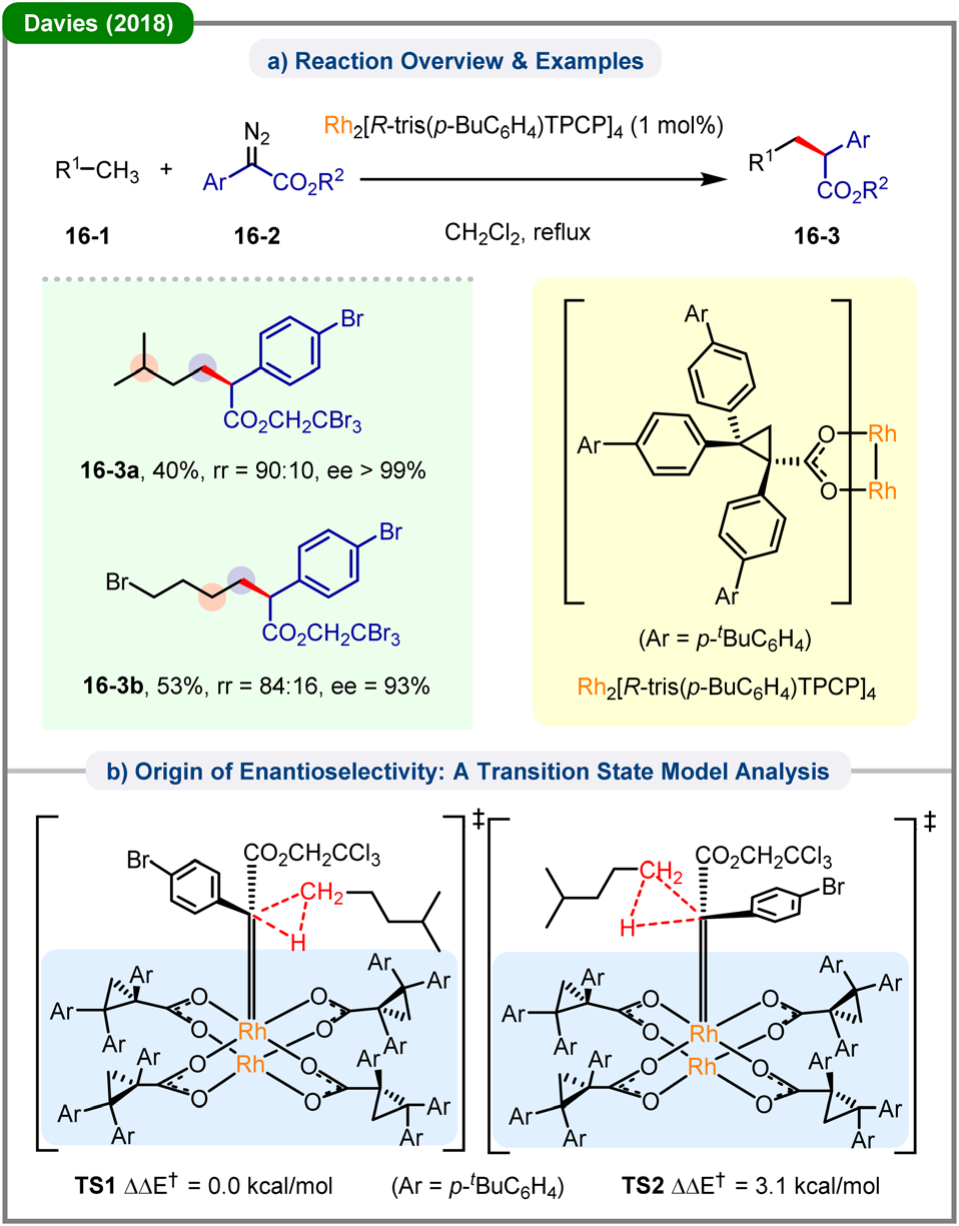
Site and enantiocontrol in primary C–H insertion using a sterically engineered dirhodium catalyst. (a) Reaction overview & examples. (b) Origin of enantioselectivity *via* a transition state model analysis.

In 2018, the steric-control principle was advanced by Davies' group through the use of a distinct class of catalysts—dirhodium tetracarboxylates (Fig. 16).^82^ The developed catalyst, Rh_2_[*R*-tris(*p-t*BuC_6_H_4_)TPCP]_4_, incorporates highly bulky triarylcyclopropane carboxylate (TPCP) ligands, generating an exceptionally congested catalytic environment, which proves highly effective for the site-selective functionalization of primary C–H bonds. For substrates such as 2-methylpentane, the catalyst directs carbene insertion toward the terminal methyl group in excellent regioselectivity (16-3a, rr = 90 : 10), achieving near-complete enantioselectivity (ee > 99%). Computational transition state analysis revealed that the chiral, pocket-like architecture of the catalyst governs the orientation of the substrate. Approach of the alkane C–H bond to one face of the carbene (leading to TS1) is sterically favored and corresponds to the lowest-energy pathway. In contrast, approach to the opposite face results in significant steric repulsion between the substrate's alkyl chain and the bulky aryl groups of the catalyst (TS2), increasing the energy by 3.1 kcal mol^−1^. This substantial energy difference between the two diastereomeric transition states establishes a strong kinetic preference, effectively channeling the reaction along a single stereochemical pathway. This work further highlights the principle that engineering highly congested catalytic environments represents a leading “Front-end Attack Control” strategy for achieving both site- and stereocontrol in challenging C(sp^3^)–H functionalization reactions.

**Fig. 17:**
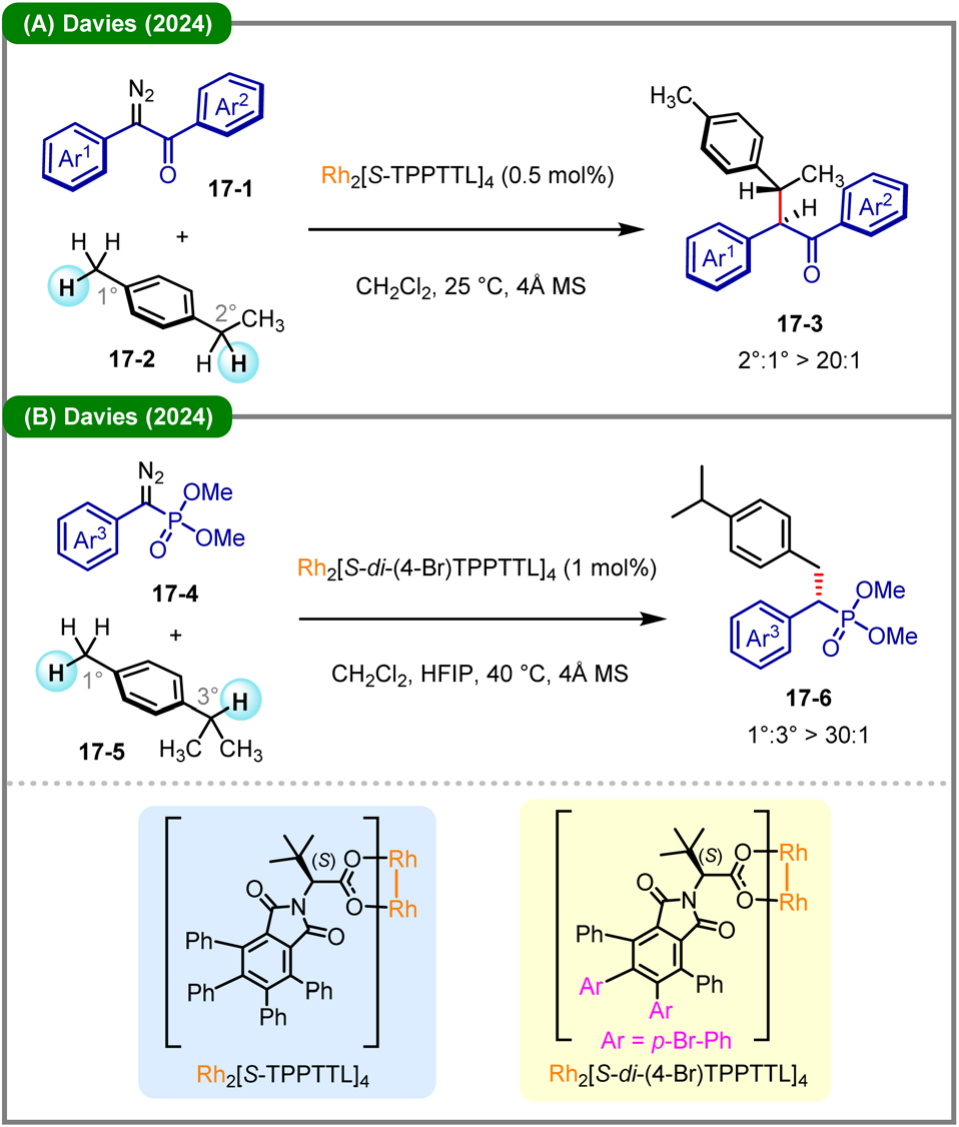
Davies' Rhodium-catalyzed enantioselective C(sp^3^)–H functionalization strategies enabled by acceptor-group tuning. (A) Approach using diaryldiazoketones as the carbene sources; (B) Approach using ((aryl)(diazo)methyl)phosphonates as the carbene precursors.

In 2024, the Davies group further extended this synthetic strategy and achieved deeper mechanistic insights into the site selectivity of diverse C(sp^3^)–H bonds *via* precise modulation of the electronic and steric properties of carbene acceptor groups. As depicted in Fig. 17, two classes of novel carbene precursors, namely diaryldiazoketones (17-1)^83^ and ((aryl)(diazo)methyl)phosphonates (17-4),^84^ were developed and coupled with optimized chiral dirhodium catalytic systems. This protocol exhibits unique selectivity profiles that differ substantially from those of conventional aryldiazoacetate-based catalysis. Specifically, the diaryldiazoketone system catalyzed by Rh_2_(*S*-TPPTTL)_4_ enables highly site-, stereo-, and diastereoselective functionalization of both activated and unactivated C–H bonds (Fig. 17A).^83^ Given its moderate steric hindrance, Rh_2_(*S*-TPPTTL)_4_ preferentially functionalizes the secondary C–H site of substrate 17-2 to afford product 17-3 (rr > 20 : 1). In contrast, the tetrahedral phosphonate moiety of precursor 17-4 imposes considerable steric bulk, which effectively switches the selectivity toward primary benzylic C–H sites of substrate 17-5 under the catalysis of Rh_2_(*S*-di-(4-Br)TPPTTL)_4_ (Fig. 17B).^84^ These advances demonstrate that the synergistic modulation of catalyst ligand scaffolds and carbene acceptor groups upgrades the selectivity regulation from early substrate-dependent paradigms to a more sophisticated catalytic engineering level. Most recently, the same group has further refined this precise selectivity control strategy. By dynamically tuning the microenvironment of catalysts to match substrate structures, the systems achieve the discriminatory functionalization of complex secondary and tertiary C(sp^3^)–H bonds, thereby mimicking the catalytic behavior of enzymatic active sites.^85,86^ These findings deepen the fundamental understanding of kinetic control in the “intermediate regime” and pave the way for the realization of superior site and stereoselectivity in C–H functionalization.

### 3.3 Control by intrinsic properties of catalysts/intermediates

While the preceding sections have focused on “Front-end” strategies that employ external physical barriers (*e.g.*, bulky ligands or molecular cavities) to kinetically block access to internal C–H bonds, terminal selectivity can also be dictated by intrinsic properties of the catalytic species itself. In such cases, the “Front-end” filtering mechanism operates not *via* steric exclusion, but rather through thermodynamic or electronic preferences inherent to the reactive intermediates. This mode of control manifests through two distinct pathways: (1) thermodynamic equilibration, wherein sterically strained internal intermediates rapidly isomerize to form more stable terminal species; and (2) intrinsic electronic bias, whereby the electrophilic character of the reagent determines the site of reaction.

#### 3.3.1 Thermodynamic selection of intermediates

In certain organometallic systems, regioselectivity is governed by the relative thermodynamic stability of metal–alkyl intermediates, rather than by the kinetic barriers associated with initial C–H activation. In these cases, C–H cleavage is typically reversible and may occur non-selectively. However, the resulting secondary or tertiary alkyl-metal species experience considerable steric strain compared to their linear, primary counterparts. As a result, rapid and reversible isomerization occurs—commonly *via* β-hydride elimination and reinsertion—leading to enrichment of the thermodynamically favored primary alkyl–metal intermediate.^11^ This equilibrated species subsequently enters the functionalization cycle. Although driven by thermodynamic factors, this approach remains within the framework of “Front-end Attack Control”: selectivity is established at the stage of metal-intermediate formation, directing reaction flux to the terminal position prior to any external trapping event. While this equilibrium-driven, thermodynamic reversibility-grounded process bears formal resemblance to the “Back-end Capture Control” regime, its underlying physical mechanism is fundamentally different. In this mode, regioselectivity arises predominantly from the relative thermodynamic stabilities of competing metal–alkyl isomers, which are mainly determined by the extent of internal steric strain relief. By contrast, it does not originate from differential kinetic rates of reaction with an external trapping agent. Within this paradigm, the external reagent functions solely as a thermodynamic sink that “locks in” the pre-equilibrated isomer distribution, rather than serving as the principal factor governing regioselectivity.

**Fig. 18:**
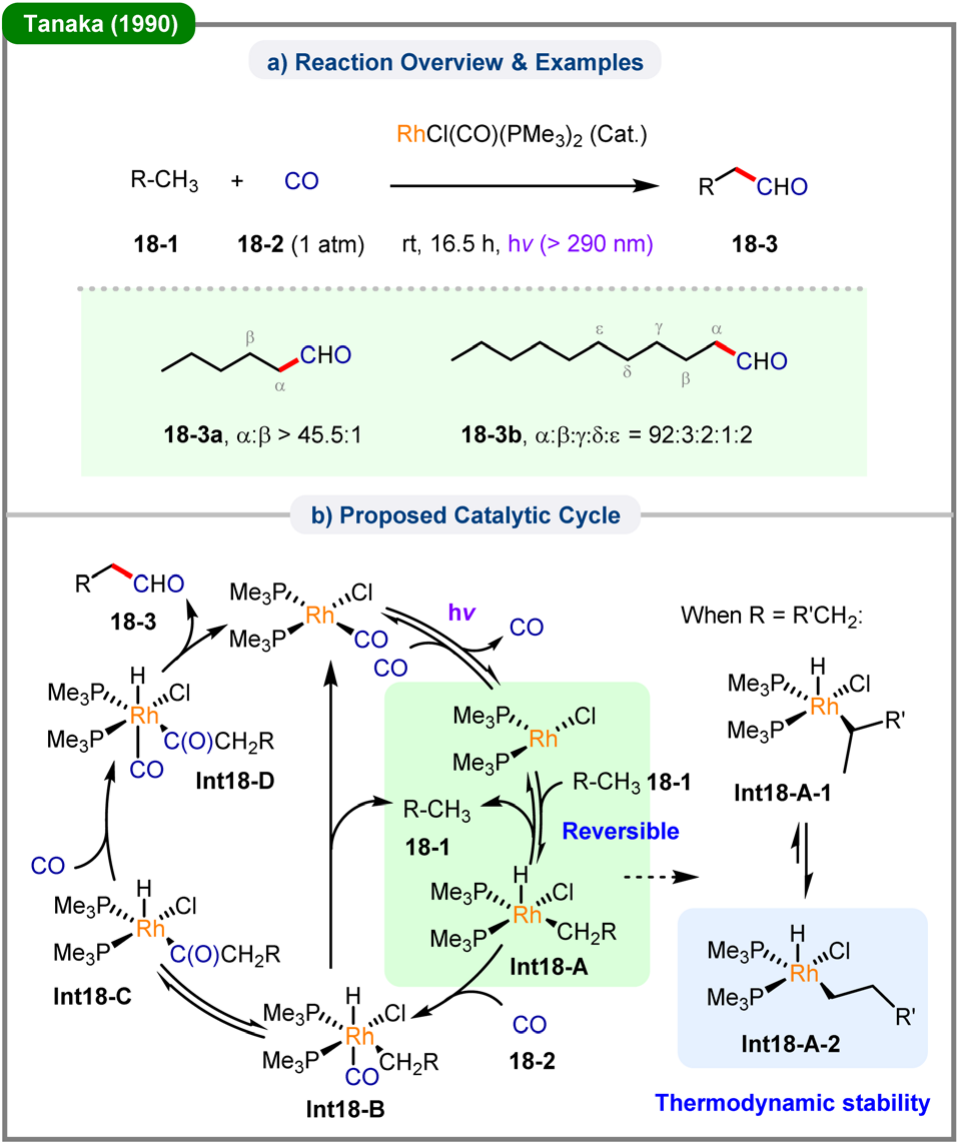
Regioselectivity control *via* thermodynamic stability of metal–alkyl intermediates. (a) Reaction overview & examples. (b) Proposed catalytic cycle.

Bennett's early work in 1978,^87^ along with Bergman's subsequent systematic studies in 1983 and 1986,^88,89^ reported alkyl isomerization resulting from the relative thermodynamic stability of reaction intermediates, thereby providing experimental support for this mechanistic principle. In 1990, Tanaka and coworkers described a photocatalytic carbonylation process that achieves highly selective terminal functionalization of linear alkanes under mild conditions with atmospheric pressure CO, using RhCl(CO)(PMe_3_)_2_ as the catalyst (Fig. 18).^90^ For example, the carbonylation of *n*-pentane demonstrated a terminal-to-internal regioselectivity exceeding 45.5 : 1, almost exclusively forming the linear aldehyde 18-3a. Importantly, this high terminal selectivity is maintained even with longer-chain alkanes (*e.g.*, 18-3b). As illustrated in the proposed catalytic cycle, photoinduced dissociation of a CO ligand generates an active rhodium species, which undergoes reversible oxidative addition into C(sp^3^)–H bonds of substrate 18-1. While both primary and secondary C–H bonds may participate in this step, a rapid isomerization equilibrium is established between the corresponding hydrido-alkyl regioisomers (*e.g.*, Int18-A). Due to its greater thermodynamic stability, the primary alkyl-rhodium complex (Int18-A-2) predominates at equilibrium over the secondary isomer (Int18-A-1). This favored intermediate is then trapped by coordination with CO, followed by migratory insertion and reductive elimination to afford the linear aldehyde product 18-3.

**Fig. 19:**
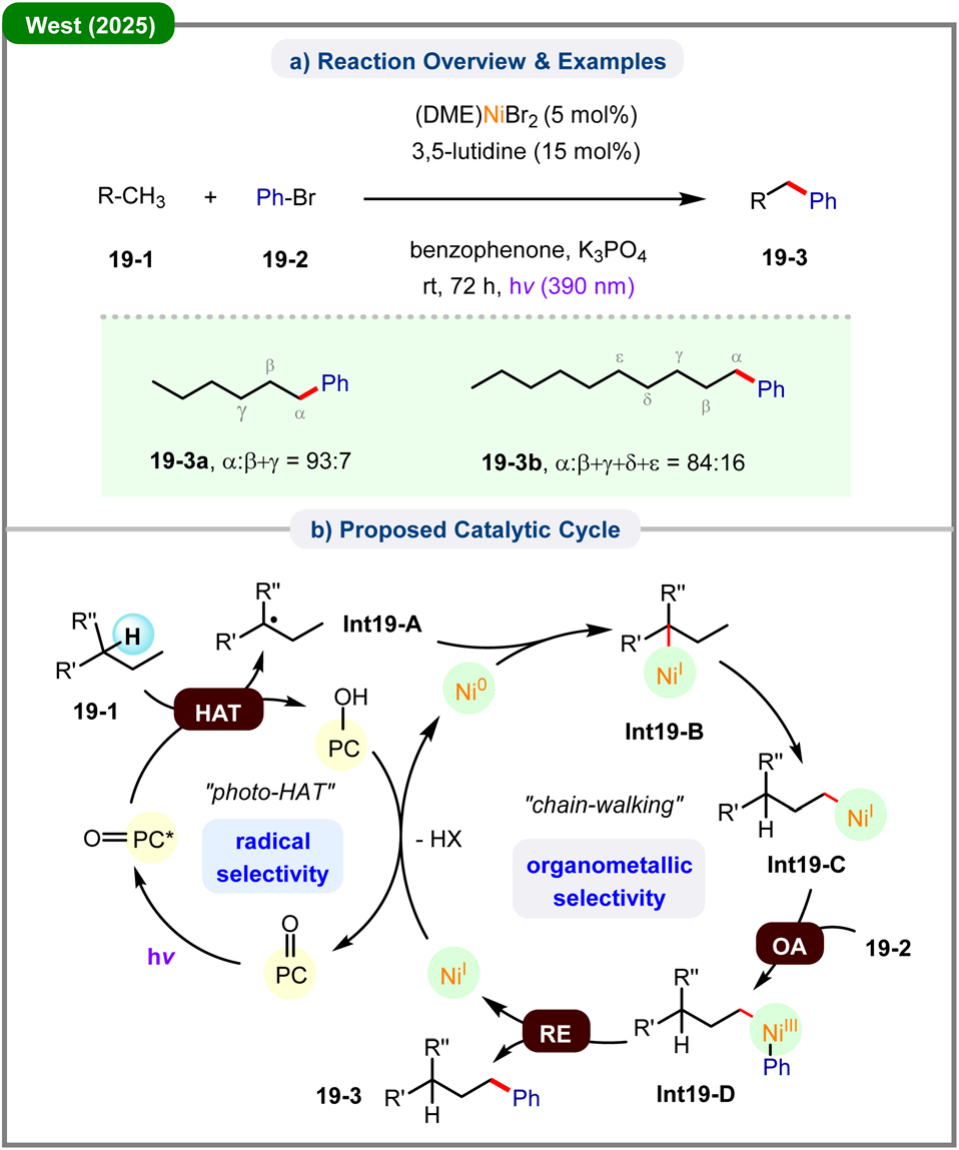
Terminal selective C(sp^3^)–H arylation *via* radical–organometallic crossover and thermodynamic selection of alkyl–nickel intermediates. (a) Reaction overview & examples. (b) Proposed catalytic cycle.

A recent illustration of this thermodynamic selection principle is embodied in the radical–organometallic crossover strategy. In 2025, the West group reported an elegant dual-catalytic system that integrates photoinduced hydrogen atom transfer with nickel-catalyzed chain-walking to achieve highly linear-selective arylation of unactivated alkanes (Fig. 19).^91^ In contrast to conventional methodologies which typically rely on sterically demanding radical traps, this approach exploits the inherent thermodynamic stability of alkyl–nickel intermediates to redirect an initially unselective radical population toward a single, terminal product. As outlined in the proposed catalytic cycle, the reaction begins with a non-selective HAT event, generating a near-statistical distribution of internal alkyl radicals. Regiocontrol is established not at this initial step, but during the subsequent radical–organometallic crossover: these transient carbon-centered radicals are rapidly intercepted by the nickel catalyst to afford sterically congested secondary or tertiary alkyl–nickel species (Int19-B). Rather than undergoing direct cross-coupling, these intermediates undergo rapid, reversible β-hydride elimination and migratory insertion, which is termed “chain-walking”. Driven by the thermodynamic preference for forming the least sterically hindered primary alkyl–nickel bond, this dynamic equilibration proceeds convergently to furnish the terminal alkyl–nickel intermediate Int19-C. Critically, the authors identified a monodentate lutidine ligand as uniquely effective in this system, as its coordinative lability enables extensive chain-walking prior to the final reductive elimination with the aryl electrophile. Here, this transformation is categorized as a “Front-end” thermodynamic control paradigm: selectivity arises neither from the initial C–H cleavage event nor from a localized steric bias at the bond-forming step (“Back-end”), but from thermodynamic equilibration of the key organometallic intermediate prior to the irreversible cross-coupling event.

#### 3.3.2 Intrinsic reactivity of active species

In contrast to strategies that rely on the external steric bulk of ligands to block internal C–H sites sterically, terminal selectivity can also be governed by the intrinsic electronic properties of the active species. In this “Front-end” regime, site discrimination is determined by the electrophilicity of the intermediate generated *in situ*—a property that can be precisely modulated through careful selection of the reagent precursor.

**Fig. 20:**
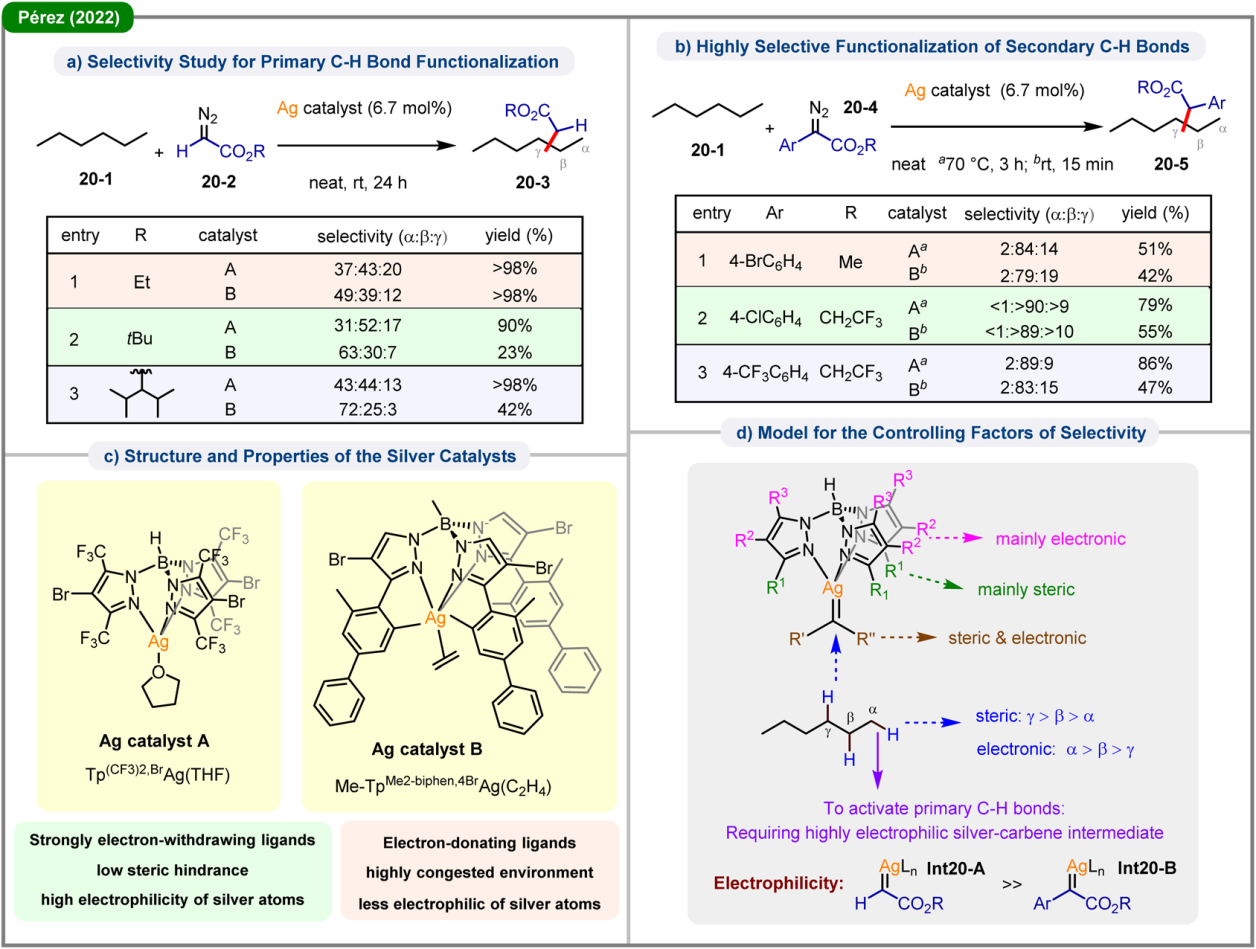
Diazo reagent-controlled regioselective functionalization of linear alkanes under silver catalysis. (a) Selectivity study for primary C–H bond functionalization. (b) Highly selective functionalization of secondary C–H bonds. (c) Structure and properties of the silver catalysts. (d) Model for the controlling factors of selectivity.

A representative example is the silver-catalyzed functionalization of linear alkanes reported by Pérez and coworkers in 2022 (Fig. 20).^92^ While the ligand environment exerts a modest influence, the primary *versus* secondary selectivity is predominantly dictated by the nature of the diazo precursor: highly electrophilic carbene precursors (*e.g.*, 20-2) favor functionalization at terminal positions, whereas stabilized donor–acceptor carbene precursors (*e.g.*, 20-4) exhibit a preference for internal sites. This “reagent-controlled” strategy contrasts sharply with the conventional “catalyst-controlled” paradigm commonly observed in rhodium-based systems, thereby offering an orthogonal electronic handle for achieving “Front-end” selectivity. The authors systematically evaluated two silver catalysts with distinct steric and electronic profiles (Fig. 20c). Catalyst A features electron-withdrawing ligands, resulting in a highly electrophilic yet minimally hindered silver center. In contrast, Catalyst B incorporates bulky, electron-donating ligands, leading to a sterically congested and less electrophilic metal environment. Notably, despite these differences, regioselectivity was primarily influenced by the electronic character of the diazo reagent rather than the catalyst structure.

As illustrated in Fig. 20d, the key factor underlying this behavior is the electrophilicity of the silver–carbene intermediate. When simple acceptor-type diazoacetates (*e.g.*, 20-2) were employed, a marked reversal in selectivity was observed, favoring terminal C–H functionalization (Fig. 20a). These precursors generate a highly electrophilic silver–carbene species (Int20-A), capable of activating the less nucleophilic but sterically accessible terminal C–H bonds. Although electronic effects dominate, steric factors can further enhance selectivity; for instance, combining a bulky ester-substituted diazoacetate with the sterically demanding Catalyst B resulted in higher selectivity for the terminal C1 position of *n*-hexane. Conversely, when donor–acceptor aryl diazoacetates (*e.g.*, 20-4) are used, the reaction exhibits exceptional selectivity for secondary C–H bonds (*β* + *γ*, > 98%) (Fig. 20b). According to the proposed model, the presence of the donor (aryl) group attenuates the electrophilicity of the silver–carbene intermediate (Int20-B). This moderately electrophilic species is sufficiently reactive to engage the more nucleophilic secondary C–H bonds but remains unreactive toward the less nucleophilic primary C–H bonds.

This silver-catalyzed system thus represents a compelling demonstration of selectivity control through the intrinsic electronic bias of the active intermediate. The findings highlight how rational design of the diazo precursor enables complete reversal of regioselectivity by fine-tuning the electrophilic character of the key intermediate. More recently, computational studies by the same group on trifluoromethyl carbenes have provided further support for this electronic-driven regiocontrol, demonstrating that a highly electrophilic carbene center can render primary C–H bond activation essentially barrierless.^93^ This principle operates independently, yet synergistically, with the steric and electronic properties of the catalyst, thereby expanding the strategic framework for achieving “Front-end” selectivity.

### 3.4 Steric control in the HAT pathway

This section outlines how sterically demanding catalyst architectures or the *in situ* formation of bulky complexes within the HAT manifold can enable selective hydrogen abstraction from terminal methyl groups through a “Front-end” control mechanism. These strategies may be broadly classified into two categories: the first leverages the inherent steric features and pre-organized microenvironments of the HAT reagents themselves, while the second adopts a dynamic approach wherein a highly reactive radical reversibly forms a sterically encumbered complex *in situ* with other system components to achieve site selectivity.

#### 3.4.1 The “microenvironment” and “intrinsic” steric effects of HAT reagents

Early investigations into the “Front-end Attack Control” strategy were largely inspired by the shape-selective catalysis exhibited by enzymes such as cytochrome P-450, prompting considerable efforts to emulate this behavior using homogeneous catalysts like metalloporphyrins.^94,95^ However, early systems employing simple, unhindered porphyrins (*e.g.*, tetraphenylporphyrin, TPP) displayed limited regioselectivity, adhering predominantly to the thermodynamic order of C–H bond strengths (tertiary > secondary > primary), thereby failing to target terminal methyl groups effectively. A natural advancement involved introducing steric bulk at the porphyrin periphery. In 1986, the Suslick group reported a seminal approach to shape-selective alkane hydroxylation using manganese porphyrins featuring deep steric “pockets”.^96^ The exceptional terminal regioselectivity control is attributed to the steric gating effect of the catalyst pocket, permitting exclusive access of the terminal methyl group to the active site, thereby shielding more reactive yet sterically hindered internal C–H bonds from oxidation.

**Fig. 21:**
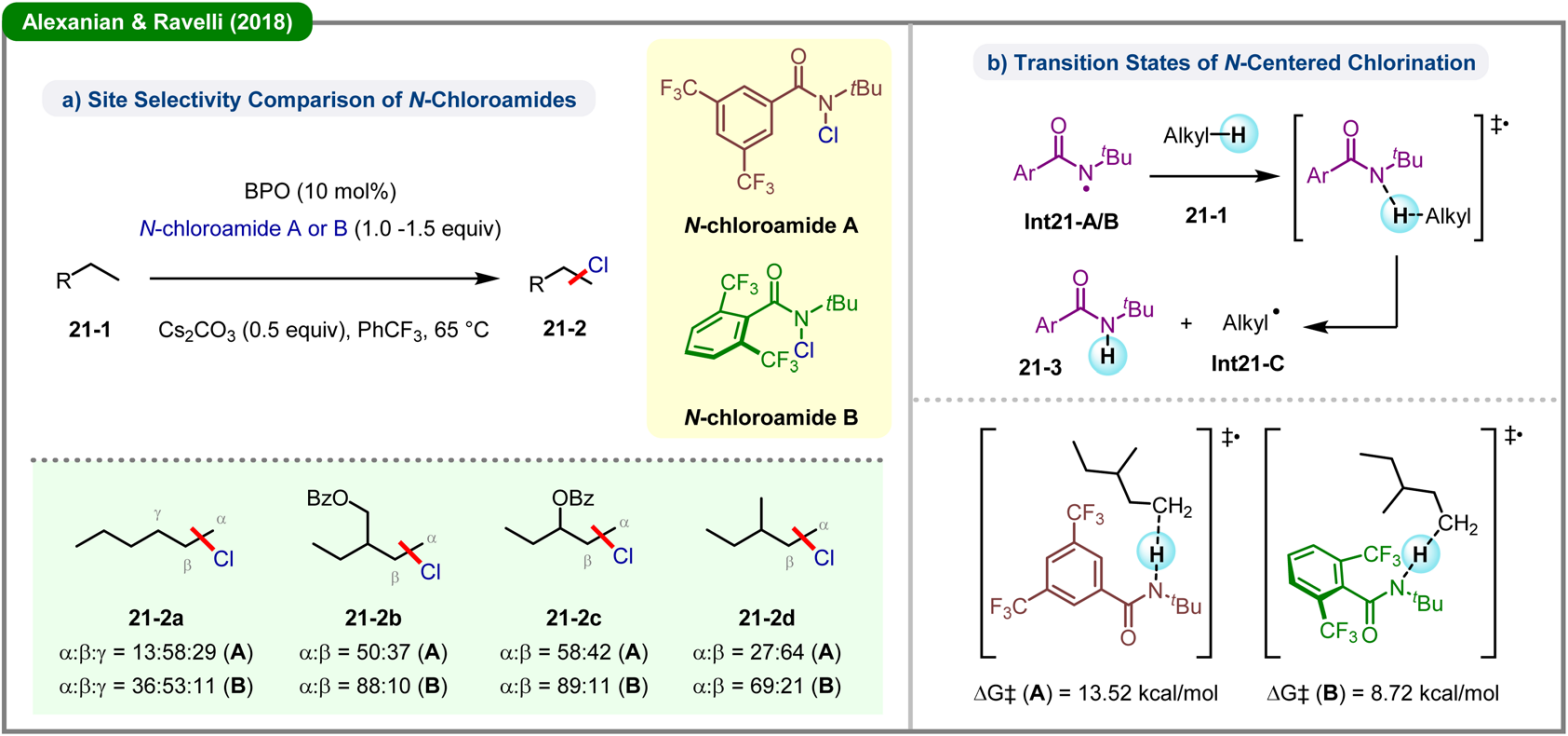
Sterically engineered *N*-chloroamides dictate terminal C–H chlorination selectivity. (a) Site selectivity comparison of *N*-chloroamides. (b) Transition states and calculated activation barriers of N-centered chlorination.

Another effective strategy involves modulating the intrinsic steric properties of the HAT reagent.^97,98^ A notable example was demonstrated by Alexanian, Ravelli, and colleagues in 2018, who designed a series of sterically hindered *N*-chloroamides to achieve reagent-controlled site selectivity (Fig. 21).^99^ The study compared a less hindered *meta*-disubstituted reagent (*N*-chloroamide A) with a sterically congested *ortho*-disubstituted analogue (*N*-chloroamide B), revealing a striking reversal in selectivity: whereas reagent A preferentially chlorinated thermodynamically weaker internal C–H bonds, reagent B favored the least hindered primary C–H sites. This switch in selectivity is rationalized by the structural conformation of the amidyl radicals (Int21-A and Int21-B, respectively): the two *ortho*-CF_3_ groups in Int21-B induce a near-perpendicular twist between the aryl ring and the amide plane, generating a highly crowded environment around the nitrogen-centered radical. DFT calculations of the transition states provide a clear kinetic rationale, showing that the activation barrier (Δ*G*‡) for hydrogen abstraction from a primary C–H bond is significantly lower for the hindered Int21-B (8.72 kcal mol^−1^) than for Int21-A (13.52 kcal mol^−1^).

**Fig. 22:**
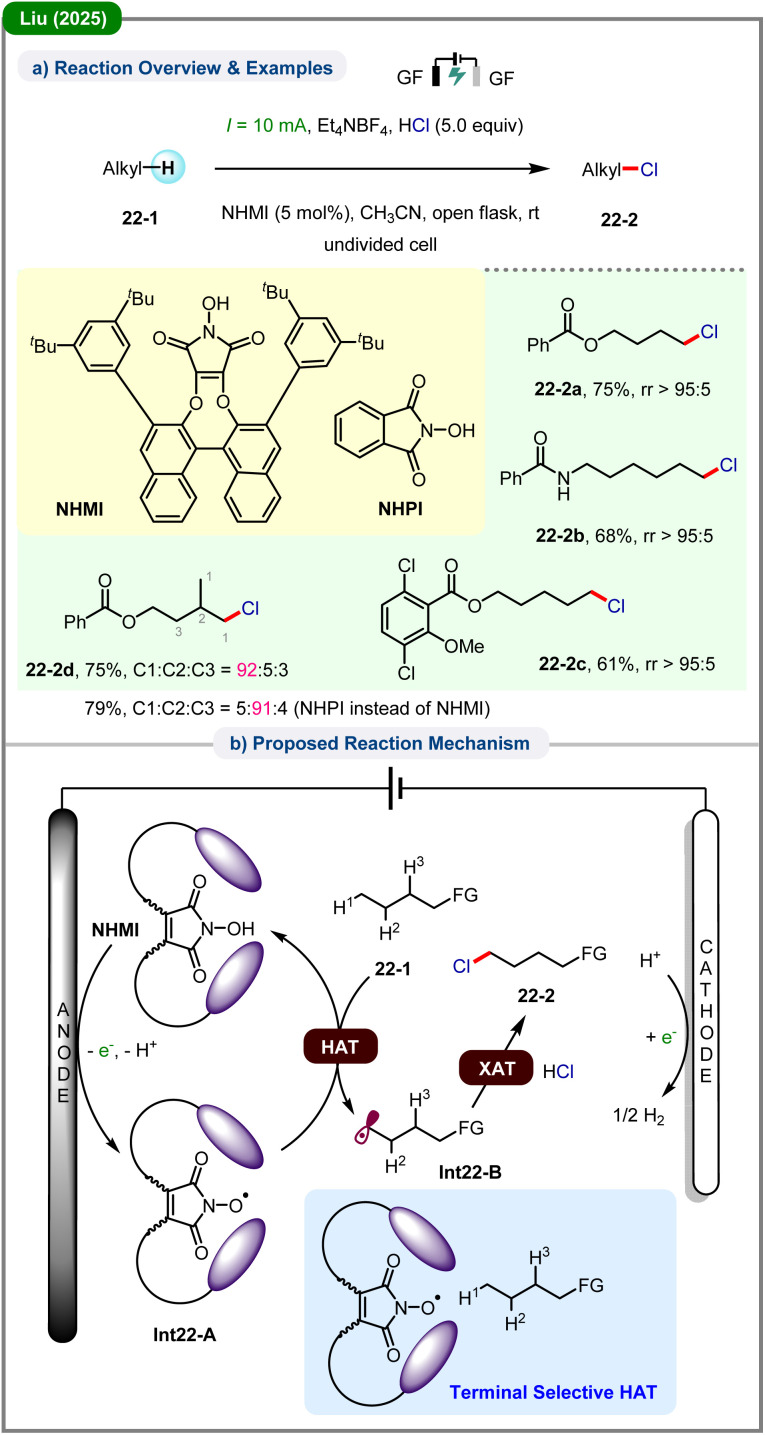
Electrochemical terminal C–H chlorination *via* a cavity-controlled organocatalyst. (a) Reaction overview & examples. (b) Proposed reaction mechanism.

Beyond modulating the intrinsic steric bulk of the HAT reagent, a more advanced strategy involves constructing a well-defined and sterically confined microenvironment around the active catalytic center. Inspired by the shape-selective recognition observed in enzymes, this approach seeks to spatially direct substrate binding such that only the least hindered terminal C–H bond can access the reactive site. A prominent example of this design principle is the recent work by Liu and colleagues, who developed an innovative electrochemical system for selective terminal C–H chlorination using cavity-controlled organocatalysts (Fig. 22).^100^ The practical efficacy of their system is evidenced by its broad substrate scope, generally affording terminal chlorinated products in good yields and with high regioselectivity (rr > 95 : 5). This level of selectivity is not governed by inherent substrate reactivity, but is instead entirely dictated by the structural architecture of the catalyst, a specially designed BINOL-derived *N*-hydroxy maleimide (NHMI).

The critical role of the pre-organized cavity is conclusively demonstrated through a control experiment (22-2d). When NHMI catalyst is employed, high terminal selectivity is achieved (C1 : C2 : C3 = 92 : 5 : 3). In contrast, replacing it with the simple *N*-hydroxyphthalimide (NHPI) completely reverses the selectivity, favoring the thermodynamically preferred internal C–H bond (C1 : C2 : C3 = 5 : 91 : 4). This stark contrast provides strong experimental support for the proposed mechanism, in which electrochemical oxidation of the NHMI catalyst at the anode generates a reactive N–O˙ radical (Int22-A) with sterically constrained cavity, which selectively accommodates the terminal methyl group of the substrate during the HAT step.

#### 3.4.2 *In situ* generated “HAT reagent-borate” complexes

A more sophisticated and dynamic “Front-end Attack Control” strategy entails the *in situ* and reversible formation of a sterically demanding complex between a highly reactive, non-selective radical and another component within the system. This transiently formed complex functions as the actual HAT reagent, leveraging its temporarily enhanced steric bulk to achieve selective control.

**Fig. 23:**
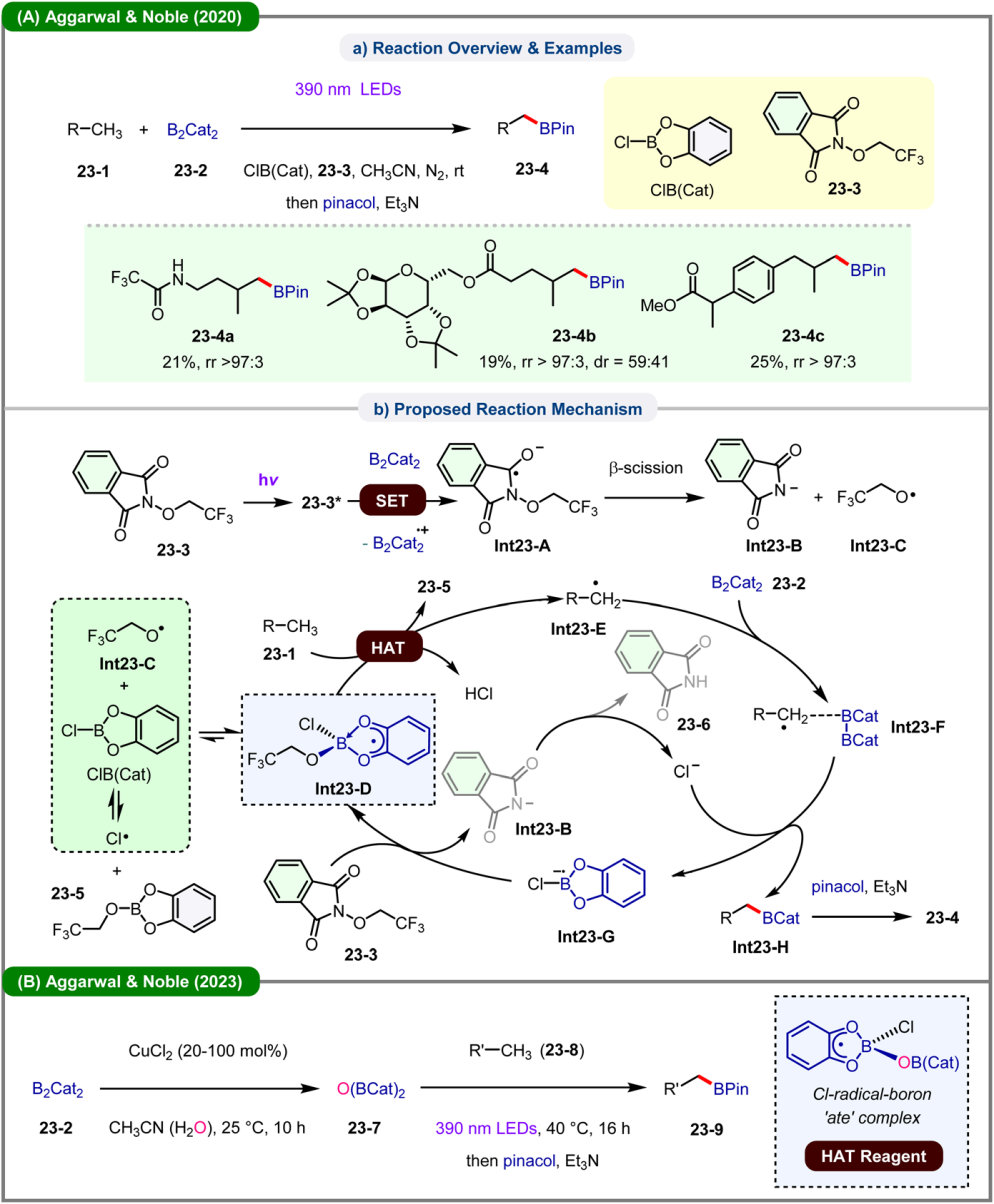
Dynamic steric control in metal-free C–H borylation *via in situ* formed borate complexes. (A) Aggarwal and Noble's 2020 work: (a) Reaction overview & examples, (b) Proposed reaction mechanism. (B) Aggarwal and Noble's 2023 work.

In 2020, a landmark study by Aggarwal and Noble on metal-free, photoinduced C(sp^3^)–H borylation presented a paradigm-shifting illustration of this approach (Fig. 23A).^101^ The reaction of alkanes (23-1) with B_2_Cat_2_ (23-2) exhibited an unusual preference for borylating stronger methyl C–H bonds over weaker secondary, tertiary, and even benzylic C–H bonds, contradicting conventional HAT principles based on BDEs. Mechanistically, the transformation is initiated by a photoinduced single-electron transfer (SET) between the *N*-alkoxyphthalimide oxidant (23-3) and B_2_Cat_2_, generating a highly reactive trifluoroethoxy radical (Int23-C). This radical does not directly participate in HAT, instead, it reversibly associates with the chloride catalyst, ClB(Cat), forming a sterically hindered chlorine radical-boron “ate” complex (Int23-D), which serves as the real HAT species. Due to its increased steric bulk, Int23-D faces significant kinetic barriers when accessing sterically congested internal C–H bonds. As a result, the reaction is kinetically directed toward the most accessible terminal methyl groups, exemplifying a refined form of “Front-end Attack Control”. Supporting evidence includes the observation that addition of a borate byproduct enhances primary selectivity, consistent with an equilibrium shift favoring the formation of the bulky “ate” complex.

In 2023, the same research group extended this principle of dynamic steric control in a copper-catalyzed dehydrogenative C(sp^3^)–H borylation system (Fig. 23B).^102^ This advancement addressed the limitation of poor atom economy inherent in the earlier protocol by eliminating the requirement for stoichiometric oxidants. A dual role was proposed for the copper catalyst, CuCl_2_: it catalyzes the reaction between B_2_Cat_2_ (23-2) and trace water to generate the electrophilic borylating agent O(BCat)_2_ (23-7); concurrently, photoinduced LMCT generates a chlorine radical, which rapidly reacts with 23-7 to form a sterically bulky chlorine radical-boron “ate” complex, as the HAT reagent selective for terminal C(sp^3^)–H borylation. Despite differing precursor pathways, both systems rely on the same fundamental “Front-end Attack Control” paradigm, wherein a dynamically generated, sterically encumbered species acts as the selective hydrogen abstractor.

## 4. “Back-end” control of site selectivity

In contrast to the “Front-end Attack Control” strategy, which governs regioselectivity during the initial C–H bond cleavage event, the “Back-end Capture Control” operates through a fundamentally distinct pathway. A critical prerequisite for this approach is a non-selective initial HAT step, for which chlorine radical (Cl˙) serves as the prototypical initiator. The distinctive nature of Cl˙ arises from its exceptionally high reactivity, exhibiting minimal discrimination among primary, secondary, and tertiary C–H bonds, and its exothermic reaction profile, leading to an “early transition state” that is largely insensitive to variations in C–H BDEs and substrate electronic effects (see Section 2.4). Upon generation, the radicals participate in a reversible intermolecular hydrogen transfer equilibrium with alkanes (R˙ + R′–H ⇌ R–H + R′˙),^103,104^ establishing a dynamic pre-equilibrium. Under the Curtin–Hammett principle, final selectivity emerges when a sterically demanding trapping agent selectively and irreversibly captures the least hindered, typically terminal, radical intermediate. Analogous to the high “cavity energy cost” (*ν*V_x_) described in the LSER model, the bulky trap imposes a significant steric barrier toward internal radicals, thereby channeling the reaction outcome predominantly through the terminal selectivity. This chapter will elaborate on various systems that implement this strategy, including those employing sterically encumbered transition-metal complexes and sterically hindered main-group radical acceptors as radical traps.

### 4.1 Transition-metal complexes as sterically demanding acceptors

Under the framework of “Back-end Capture Control”, transition-metal complexes function as efficient kinetic interceptors. By engineering a congested ligand environment around the metal center, the system can preferentially capture terminal radicals from an otherwise non-selectively generated radical pool. The feasibility of this logic was demonstrated by Hartwig and coworkers in their studies on copper-catalyzed alkane amidation;^11^ in this manifold, the *in situ*-generated, sterically demanding copper complex effectively filters out more hindered internal radicals (refer to Section 5.2 for a discussion on the mechanistic boundaries of this system with specific substrates).

**Fig. 24:**
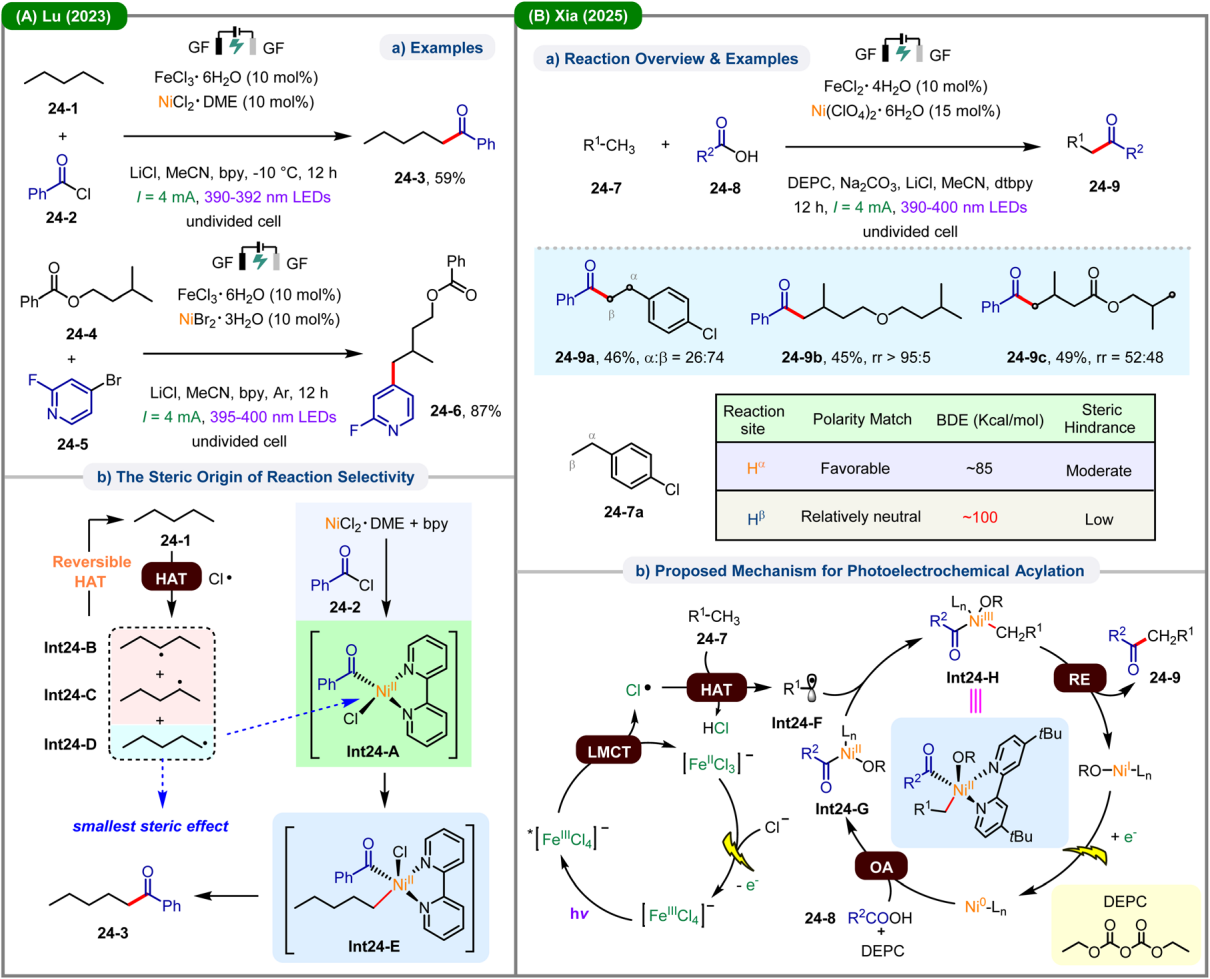
Achieving terminal selectivity *via* “Back-end Capture Control” in photoelectrochemical iron–nickel dual catalysis. (A) Lu's works: (a) examples, (b) steric origin. (B) Xia's work: (a) reaction overview & examples, (b) proposed mechanism.

The advancement of modern photoelectrocatalytic technologies has enabled the development of paired systems that integrate oxidative and reductive transformations within a single electrolytic cell.^105^ A representative example of this approach is the iron–nickel dual catalytic platform, which exemplifies the application of such integrated systems to C(sp^3^)–H functionalization.^106–108^ In 2023, Lu and colleagues demonstrated the utility of this system in C–H acylation and arylation reactions under mild photoelectrocatalytic conditions (Fig. 24A).^106,107^ The observed terminal selectivity in these transformations can be rationalized by the “Back-end Capture Control” mechanism. Following an initial, non-selective HAT step that generates a near-statistical mixture of alkyl radicals, selective functionalization is achieved through trapping by a sterically demanding organonickel species (Int24-A). This intermediate exhibits a strong kinetic preference for less hindered terminal radicals (*e.g.*, Int24-D) due to significant steric constraints, while effectively suppressing reaction pathways involving more congested internal radicals (*e.g.*, Int24-B and Int24-C).

Further experimental support for this steric control model is provided by Xia's C(sp^3^)–H acylation system (Fig. 24B).^108^ Notably, in the functionalization of 4-chloroethylbenzene (24-7a), two competing sites offer an instructive comparison: the benzylic α-position possesses favorable thermodynamic characteristics (BDE ≈ 85 kcal mol^−1^) and electronic properties conducive to polar matching, whereas the terminal β-position is favored only on the basis of steric accessibility. Despite the significant thermodynamic and electronic advantages of the α-site, product 24-9a was obtained with predominant β-selectivity (*α* : *β* = 26 : 74). This outcome underscores that steric effects in the “Back-end” radical capture step serve as the dominant factor governing regioselectivity, capable of overriding both thermodynamic and electronic biases. The proposed mechanism contains two interdependent cycles: in the anodic cycle, photoexcitation of a Fe(iii) complex *via* LMCT generates Cl˙ to mediate HAT from unactivated C(sp^3^)–H bonds; forming an alkyl radical (Int24-F); concurrently, the cathodic cycle involves the reductive formation of a Ni(0) complex, which undergoes oxidative addition with an *in situ*-generated mixed anhydride (by reacting 24-8 with diethyl pyrocarbonate, DEPC) to yield a sterically encumbered acyl-Ni(ii) complex (Int24-G), acting as the key radical trap. This species captures transient alkyl radical Int24-F to form a high-valent Ni(iii) intermediate (Int24-H), which subsequently undergoes reductive elimination to afford the ketone product (24-9).

### 4.2 Sterically demanding main-group radical acceptors

Another mechanistically unique yet conceptually related approach relies on single earth-abundant metals—especially iron—and sterically encumbered main-group radical acceptors to enable “Back-end Capture Control”. From the standpoint of the LSER model, this approach relies on the high energetic cost associated with cavity formation during the transition state. Bulky main-group reagents based on sulfur, phosphorus, or boron create a rigid microenvironment during the capture step. The incorporation of a sterically congested tertiary radical into this constrained space necessitates substantial cavity expansion, resulting in a prohibitive energetic penalty (*ν*V_x_). This steric demand significantly elevates the activation barrier (Δ*G*‡) for the internal pathway, thereby directing the dynamic equilibrium of radical intermediates exclusively toward the less hindered terminal radical, in accordance with the Curtin–Hammett principle.

This steric-gating strategy is effectively implemented within the photoinduced LMCT framework, as demonstrated by the Jin and Duan's pioneering work on iron-photocatalyzed alkynylation.^47^ Its mechanistic rationale was initially attributed primarily to the steric properties of alkynyl sulfone acceptors—a principle that, as outlined in Section 2.4, forms the foundation of the “Back-end Capture Control” model. Building upon this conceptual basis, subsequent studies by multiple research groups have systematically established that this approach represents not an isolated case, but rather a general and robust strategy. Its applicability has been extended to a broad range of C(sp^3^)–H functionalization reactions, including borylation, thiolation, sulfinylation, and phosphonylation.

**Fig. 25:**
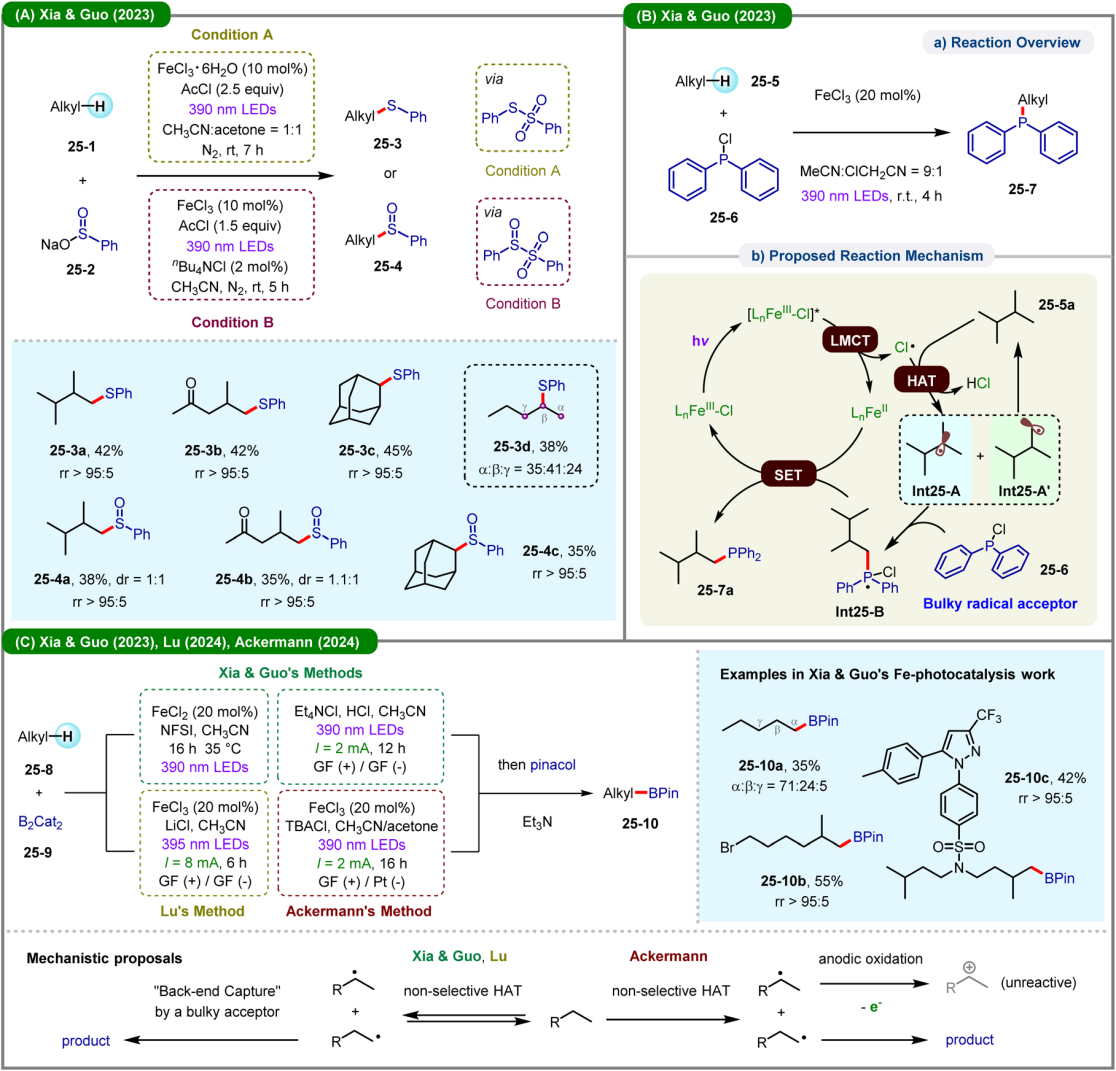
Iron-photo(electro)catalyzed terminal C–H thiolation, sulfinylation, phosphorylation, and borylation by “Back-end Capture Control”. (A) C–H thiolation and sulfinylation; (B) C–H phosphorylation; (C) C–H borylation.

A clear illustration of this strategy can be provided by Xia and Guo's 2023 report on iron-catalyzed C–H thiolation and sulfinylation (Fig. 25A).^109^ Highly reactive chlorine radicals are generated *via* photoinduced LMCT, undergoing non-selective HAT with alkanes to produce a near-statistical distribution of alkyl radicals. Regioselectivity is thus determined exclusively at the subsequent radical capture step: the interception of these radical intermediates by *in situ*-generated, sterically demanding sulfur-based acceptors. This steric-controlled kinetic filtration is particularly evident in structurally congested substrates. For isobutyl methyl ketone, exceptional terminal selectivity (rr > 95 : 5) is observed (25-3b). Similarly, in the case of rigid adamantane, functionalization occurs exclusively at the less hindered secondary (β) positions (25-3c). In both cases, the substantial steric barrier imposed by the trapping agent effectively suppresses pathways involving internally substituted, sterically encumbered radicals. Nevertheless, this control regime exhibits obvious limitations in the functionalization of flexible linear alkanes, as exemplified by *n*-pentane (25-3d, *α* : *β* : *γ* = 35 : 41 : 24). The marginal steric differences between terminal and internal C–H sites prevent the bulky trapping species from establishing dominant kinetic selectivity. Accordingly, the product profile reflects a balance of competing effects. It highlights that the performance of “Back-end Capture Control” is fundamentally governed by the combined effects of reversible HAT and the steric selectivity of the radical acceptor.

In the same year, the same group further expanded this methodology to C(sp^3^)–P bond formation (Fig. 25B).^110^ This strategy employs chlorodiphenylphosphine (25-6) as a sterically bulky radical trap, achieving excellent terminal selectivity for branched alkanes. The catalytic cycle also illustrates the operation of the “Back-end Capture Control” model: steric effects govern the divergent fates of primary (Int25-A) and tertiary (Int25-A′) alkyl radicals generated *via* HAT: the bulky reagent 25-6 selectively captures the accessible primary radical (Int25-A) to form the P-centered radical intermediate Int25-B. In contrast, the more sterically hindered tertiary radical (Int25-A′) is kinetically disfavored for capture and instead undergoes reversible HAT with the parent alkane (25-5a), re-entering the equilibrium pool. This dynamic pre-equilibrium effectively channels the reaction flux toward the less hindered terminal position, enabling high levels of regioselectivity. Exploiting iron-photocatalysis, the groups of Hu and Jia,^111^ Huang and Zhu^112^ also independently reported their systems for direct C(sp^3^)–P bond construction, with terminal selectivity observed in certain cases.

This principle of employing sterically demanding main-group radical acceptors finds its most extensive application in the context of C(sp^3^)–H borylation.^113^ Several groups, including those of Xia and Guo,^109,114^ Lu,^115^ and Ackermann,^116^ have developed distinct iron-catalyzed photo(electro)chemical systems that achieve remarkable terminal selectivity (Fig. 25C). Taking the photocatalytic system reported by Xia and Guo^109^ as an example, excellent terminal selectivity (rr > 95 : 5) was observed across a range of sterically differentiated substrates, including complex functionalized molecular scaffolds. However, selectivity also diminishes with linear alkanes such as *n*-pentane, which yields a product mixture favoring the terminal position but still containing appreciable amounts of internal isomers (25-10a, *α* : *β* : *γ* = 71 : 24 : 5). Notably, although the experimentally observed selectivity patterns are broadly similar among these iron-based systems, the proposed mechanistic rationales differ. Xia and Guo's group,^109,114^ and Lu's group^115^ attributed the preference for terminal functionalization to a “Back-end Capture Control” mechanism: the bulky borylating agent B_2_Cat_2_ selectively intercepts the less hindered primary radicals from an equilibrated radical pool. In contrast, the Ackermann group proposed another pathway for their photoelectrochemical system.^116^ According to this proposal, following a non-selective HAT step, the thermodynamically more stable internal radicals—which are also more readily oxidized—are selectively removed *via* anodic overoxidation, forming unreactive carbocations. This process effectively depletes the internal radical species, allowing the less oxidizable primary radicals to be captured by the borylating agent, thereby establishing terminal selectivity.

## 5. Beyond the binary: mechanistic ambiguities, alternative pathways, and synergistic control

The binary classification into “Front-end Attack Control” and “Back-end Capture Control” provides a robust logical framework for rationalizing terminal selectivity, yet it represents an idealized simplification of a more multifaceted kinetic reality. In practice, regioselectivity is not always governed by a single kinetic bottleneck. Instead, it can arise from continuous, spatially distributed kinetic flux across multiple catalytic cycles, as demonstrated by synergistic relay catalysis (Section 5.1). Furthermore, these kinetic gating effects have inherent limitations. Catalyst-derived steric filtering often reaches a performance ceiling when competing with strong substrate-intrinsic electronic or thermodynamic preferences, thereby compromising regiocontrol (Section 5.2). Additionally, photoelectrochemical systems introduce another dimension of unexpected selectivity deviation, in which anodic oxidation of intermediates opens alternative pathways that further reshape the regiochemical outcome (Section 5.3). By integrating such synergistic, limiting, and pathway-convergent scenarios, this chapter transcends the conventional binary paradigm and delineates the intrinsic complexity governing state-of-the-art C(sp^3^)–H functionalization.

### 5.1 “Synergistic control” in relay catalysis

Beyond the conventional binary model lies a distinct class of systems that resist assignment to a single kinetic gating step. In these systems, site-selectivity cannot be adequately described as either “filtering” at the initiation stage or “capture” in downstream steps. Rather, they exemplify “synergistic control”: a mode wherein selectivity emerges from the concerted rate matching of multiple discrete elementary steps across the full catalytic cycle, enabled by a “kinetic funneling” effect.

**Fig. 26:**
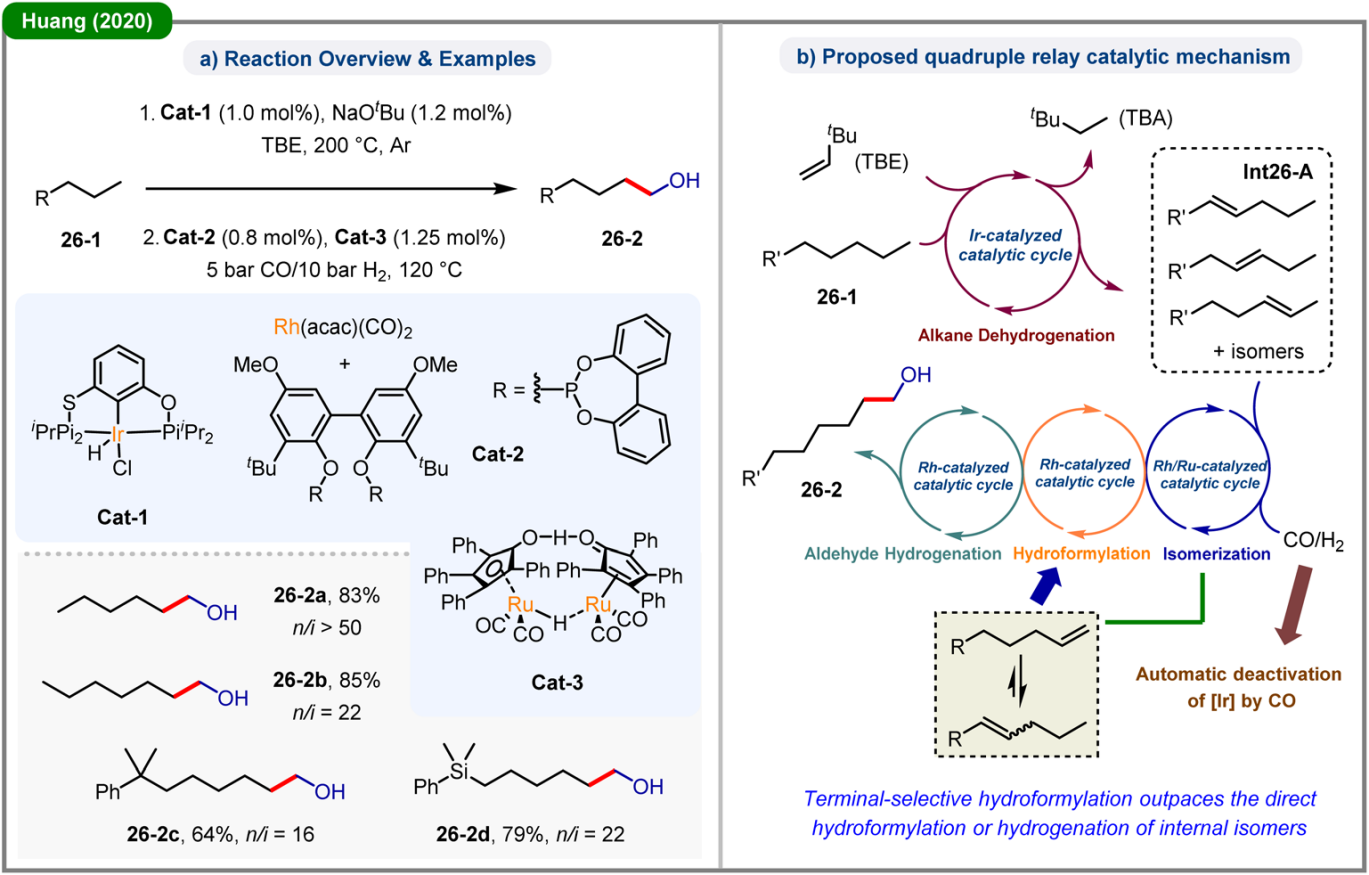
Terminal-selective C(sp^3^)–H hydroxymethylation *via* quadruple relay catalysis involving kinetic flux funneling and dynamic catalyst. (a) Reaction overview & examples. (b) Proposed quadruple relay catalytic mechanism.

A representative demonstration of this principle is Huang's 2020 quadruple relay catalytic system (“AD-ISO-HF-AH”), which enables the formal hydroxymethylation of linear alkanes (Fig. 26).^117^ The initial iridium-catalyzed alkane dehydrogenation (AD) proceeds in a non-selective manner, generating a thermodynamically governed mixture of internal alkene intermediates (Int26-A). This non-discriminatory reactivity excludes the AD step from the regime of Front-end Attack Control. Notably, the sequential cascade of alkene isomerization (ISO), hydroformylation (HF), and hydrogenation (AH) establishes a dynamic selectivity-filtering mechanism. Critically, terminal alkenes undergo hydroformylation orders of magnitude more rapidly than their internal counterparts. This strong kinetic preference continuously channels the reaction flux toward terminal functionalization, affording excellent terminal selectivity with an *n*-/*i*-alcohol ratio of up to 50 : 1.

Furthermore, this relay system features dynamic catalyst evolution behavior. Following dehydrogenation, the active iridium catalyst (Cat-1) spontaneously transforms into inert carbonyl adducts under syngas conditions. Such catalyst deactivation effectively suppresses off-cycle side reactions—including unselective alkene hydrogenation in downstream steps—thereby preserving the selectivity established by the relay cascade throughout the catalytic process. Under this “synergistic control” mode, terminal selectivity does not originate from a single selectivity-determining transition state; instead, it arises from sustained, spatially distributed kinetic flux throughout the multi-metallic catalytic cascade.

It is important to clarify that this quadruple relay catalysis proceeds *via* alkene intermediates rather than direct σ-bond activation or radical C(sp^3^)–H functionalization, rendering the transformation a “formal” C(sp^3^)–H hydroxymethylation. Nevertheless, its inclusion in this discussion is conceptually indispensable. When conventional “Front-end” or “Back-end” gating strategies fail to achieve exclusive terminal selectivity for complex unactivated alkanes, this relay paradigm offers an unconventional alternative: selectivity regulation is no longer confined to a single reaction step but is redistributed as a cooperative kinetic funnel spanning multiple interconnected catalytic cycles.

### 5.2 Counter-examples: when steric control fails

**Fig. 27:**
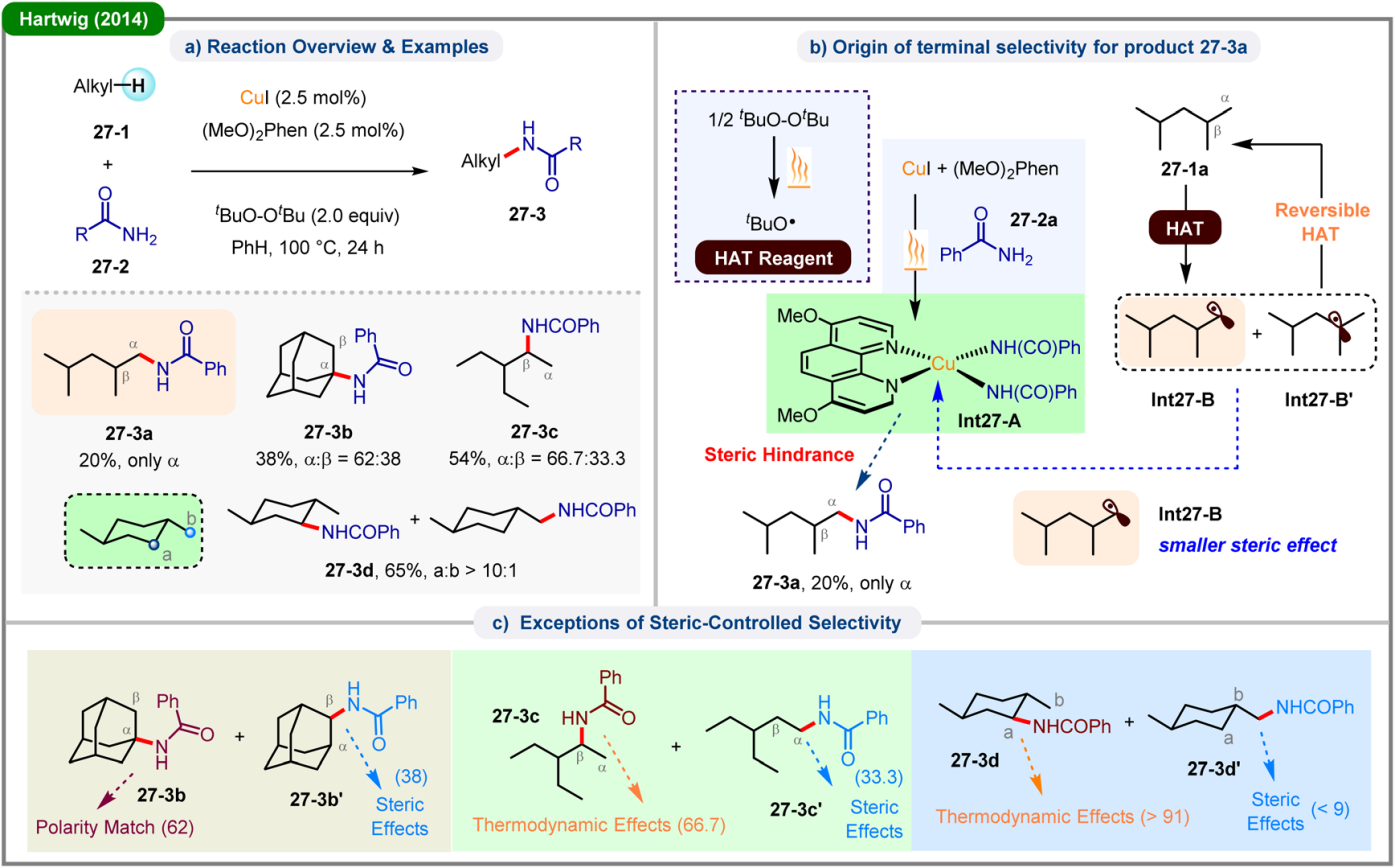
Interplay between polarity-match and steric-hindrance in Hartwig's copper-catalyzed C(sp^3^)–H amidation. (a) Reaction overview & examples. (b) Origin of terminal selectivity for product 27-3a. (c) Exceptions of steric-controlled selectivity.

Although the binary distinction between “Front-end Attack Control” and “Back-end Capture Control” provides a robust predictive framework, its validity can be challenged by intrinsic substrate electronic or thermodynamic properties that override catalyst-imposed steric gates. The copper-catalyzed C(sp^3^)–H amidation developed by Hartwig and co-workers (Fig. 27)^11^ represents a quintessential example of this mechanistic boundary. In this manifold, the bulky, *in situ*-generated copper(ii) complex (Int27-A) typically exerts a strong “Back-end Capture Control”: less-hindered radicals, such as the primary radical Int27-B, recombine with the copper center at rates significantly higher than those of the congested internal radicals (Int27-B′). Such steric exclusion directs functionalization of intrinsically hindered substrates (*e.g.*, 2,4-dimethylpentane) exclusively to the primary position (27-3a).

However, this logic undergoes a pronounced dynamic shift when applied to substrates bearing substantial electronic or thermodynamic disparities (Fig. 27c). For adamantane, a potent polarity-driven override prevails despite the considerable steric bulk of the catalyst: optimal alignment between the electrophilic radical species and the electron-rich tertiary (3°) C–H bond at the bridgehead (α) site effectively bypasses steric filtration, yielding a 3° product ratio as high as 62% (27-3b). Moreover, in the amidation of 3-ethylpentane (27-3c) and *trans*-1,4-dimethylcyclohexane (27-3d), the lower BDE of secondary (2°) C–H bonds manifests as a significant thermodynamic driving force that overrides steric repulsion, allowing the secondary product to predominate. Together, these observations reveal a critical principle: if the “Back-end” radical capture fails to establish an absolute kinetic lock, the overall regioselectivity will spontaneously drift toward thermodynamically and electronically favored C–H positions.

**Fig. 28:**
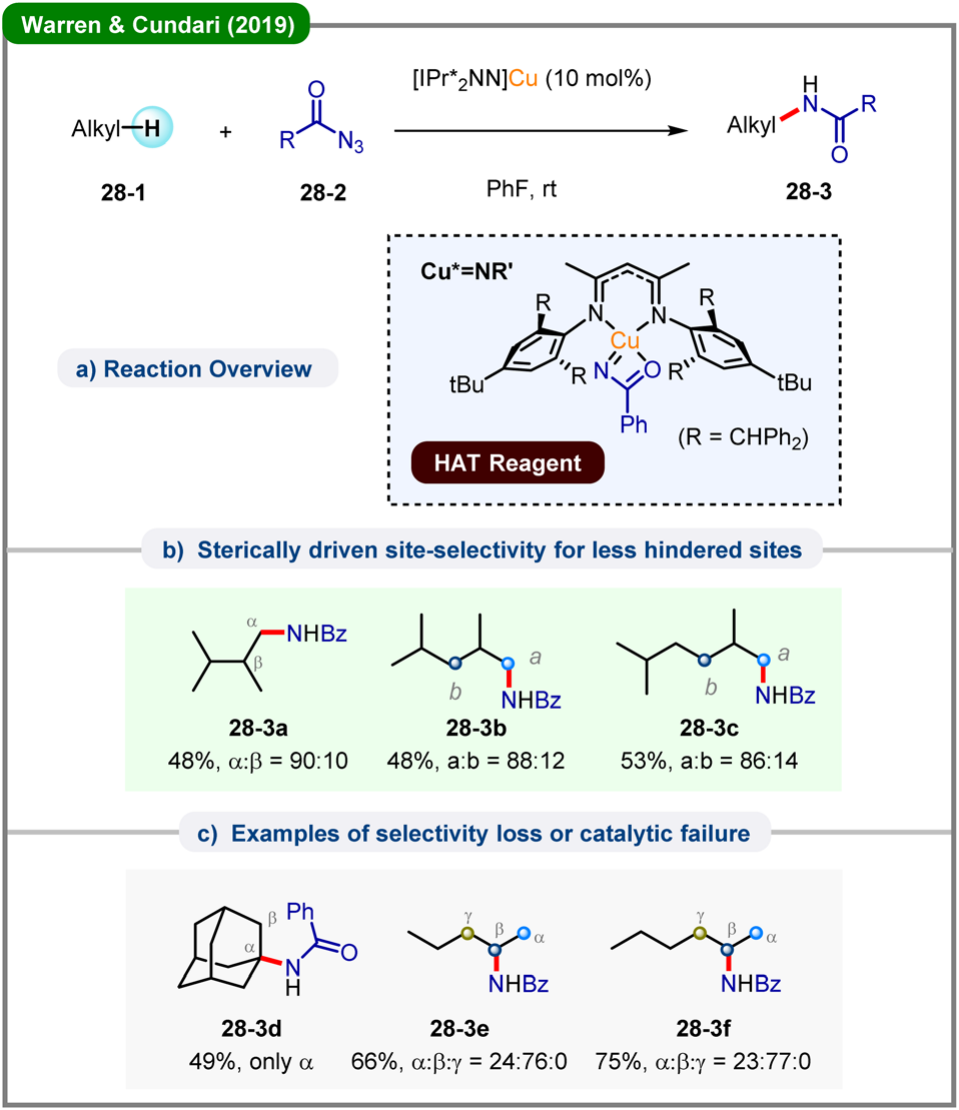
Persistence of substrate-dictated selectivity in Warren and Cundari's copper-catalyzed C(sp^3^)–H amidation despite extreme ligand bulk. (a) Reaction overview. (b) Sterically driven site-selectivity for less hindered sites. (c) Examples of selectivity loss or catalytic failure.

The work by Warren and Cundari's group (Fig. 28)^118^ provides additional evidence for the above principle, showcasing that bulky catalyst frameworks lose their governing power in the presence of strong substrate electronic and thermodynamic biases. The authors designed β-diketiminate ligands featuring four *ortho*-di(benzhydryl) (*o*-CHPh_2_) substituents, constructing a highly encumbered coordination environment for the copper–nitrene intermediate to enforce selective amidation at primary and secondary C–H bonds. While this system achieves highly selective terminal methyl amidation for branched alkanes (28-3a – 28-3c), it fails to maintain such selectivity for substrates with prominent site-specific reactivity. For example, adamantane undergoes exclusive amidation at the bridgehead tertiary position (28-3d), as optimal polarity interaction between the electrophilic nitrene and electron-rich tertiary C–H bonds dominates over steric effects. For flexible linear alkanes, C2-selective functionalization is observed (28-3e, 28-3f), originating from a thermodynamically favorable transition state with reduced energy. It is therefore clear that the “Front-end” steric tuning also has fundamental constraints. Strong electronic and thermodynamic effects originating from the substrate will ultimately prevail over steric repulsion.

By integrating these counter-examples in competitive scenarios, our binary framework evolves from a rigid classification into a dynamic spectrum of competing kinetic influences.

### 5.3 Photocatalysis under electrochemical conditions: more complex exceptions to selectivity

**Fig. 29:**
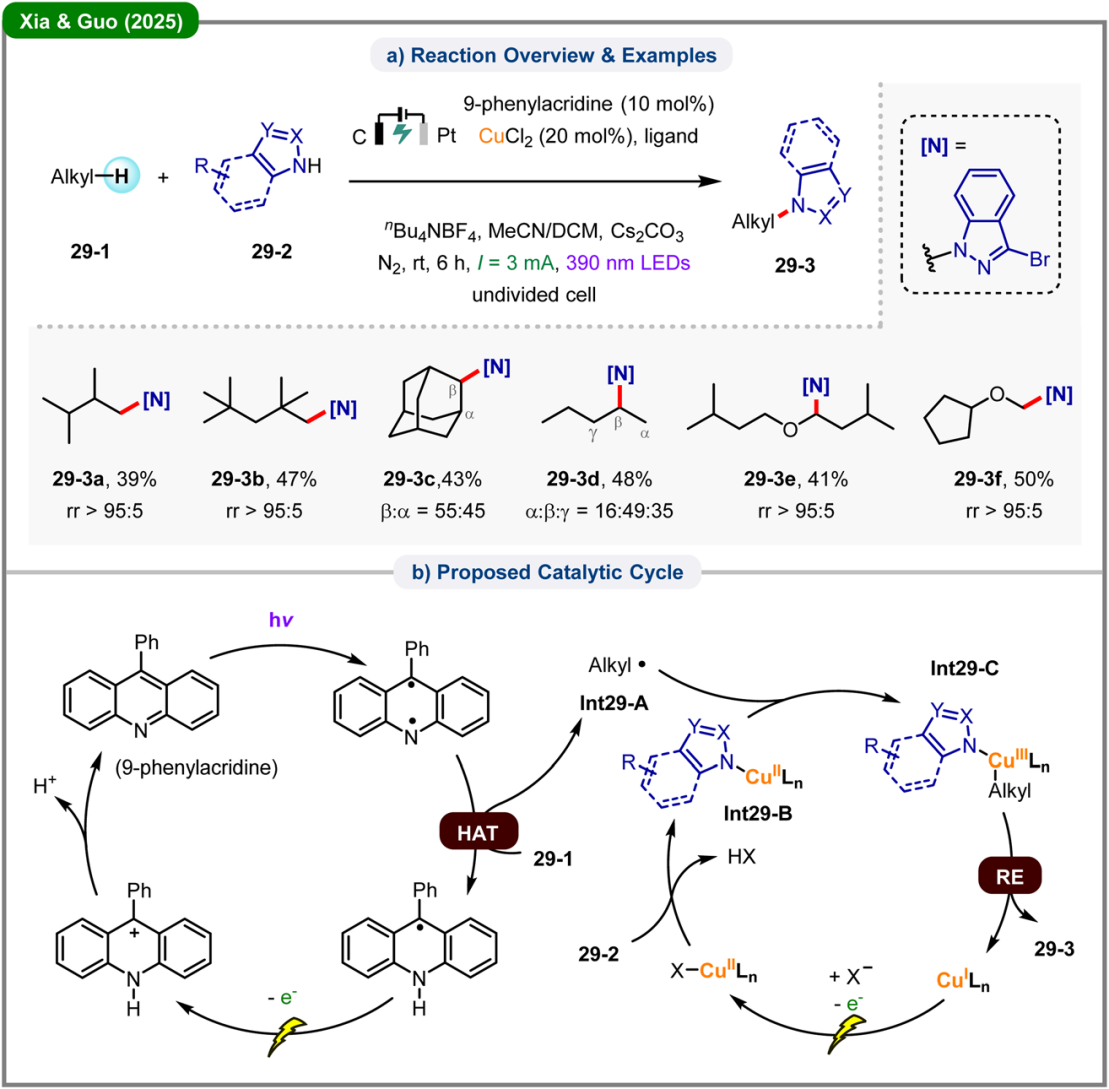
Photoelectrochemical acridine/copper dual-catalyzed C(sp^3^)–H functionalization. (a) Reaction overview & examples. (b) Proposed catalytic cycle.

Analogous to the anomalous reactivity trends described in Section 5.2, photoelectrochemical reactions also display distinct irregular selectivity behaviors. While such anomalies tend to be more complex and governed by synergistic regulatory effects, the photoelectrochemical dual-catalytic system recently developed by Xia, Guo, and coworkers serves as a representative prototype of this synergistic regulation mode (Fig. 29).^119^ This work integrates 9-phenylacridine as a direct photo-HAT reagent with a copper-mediated catalytic cycle to establish a “dual-filtration” platform, whose selectivity exhibits pronounced substrate-dependent modulation. For highly branched 2,3-dimethylbutane, the sterically encumbered 9-phenylacridine biradical experiences significant repulsion from the shielded tertiary (3°) C–H sites. As a result, steric discrimination dominates at the “Front-end” HAT step, affording exceptional terminal selectivity (29-3a). In contrast, with linear alkanes exhibiting minimal steric differentiation such as *n*-pentane, the efficacy of this steric gate diminishes markedly. Here, the lower BDEs of internal (2°) C–H bonds enable thermodynamic and electronic factors to reassert dominance, shifting selectivity toward internal functionalization (29-3d). Notably, the high α-selectivity observed with electron-rich substrates such as ethers (*e.g.*, 29-3e and 29-3f) reveals an additional layer of mechanistic complexity. Although polarity-matched HAT remains a plausible contributor, an alternative electrochemical pathway cannot be excluded: anodic oxidation of the substrate generates a radical cation, which undergoes rapid deprotonation to yield a carbocation intermediate; this electrophilic species is then intercepted by nucleophilic reagents. Such an electro-oxidation pathway (also implicated in iron-based photoelectrocatalytic system by Ackermann's group^116^) yields product distributions that closely mirror those of the radical-mediated route. Consequently, distinguishing the operative kinetic control center remains experimentally challenging.

Such mechanistic uncertainty necessitates a refined interpretation of the binary framework proposed in this review. Within the intermediate regime, it is essential to systematically evaluate how key operational parameters—including electrode potential, reagent steric properties, and electrolyte composition—dynamically reshape the reaction coordinate. The integration of these competing pathways and boundary cases upgrades our original framework from an idealized static model to a condition-adaptive, analytically quantitative tool. This revised paradigm accommodates the complex kinetic realities of contemporary catalytic systems and provides generalizable design principles for the precision construction of C–H functionalization platforms.

## 6. Conclusions and outlook

**Fig. 30:**
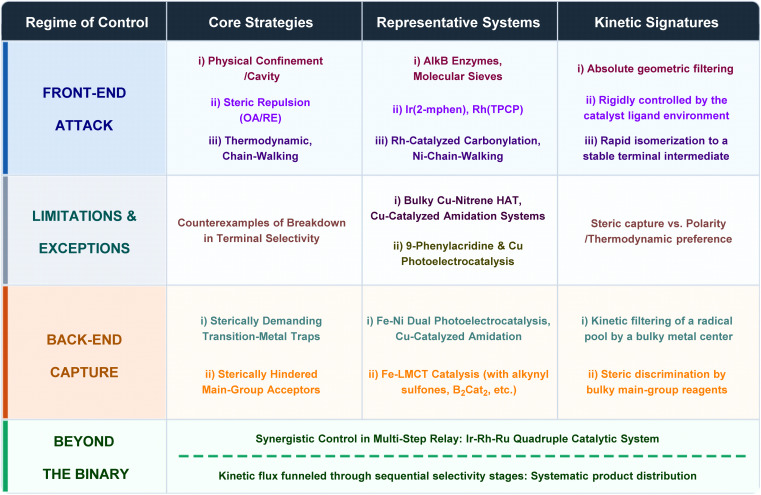
Summary and comparison of kinetic control paradigms for terminal-selective undirected C(sp^3^)–H functionalization: strategies, representative systems, and kinetic signatures.

This review systematically analyzes the kinetic origins of terminal-selective C(sp^3^)–H functionalization (Fig. 30). Two principal mechanistic models, “Front-end Attack Control” and “Back-end Capture Control”, are classified according to the kinetic stage where terminal selectivity is predominantly established, which are applied to rationalize the majority of existing approaches. Importantly, terminal selectivity is not a fixed intrinsic property but a dynamic result of kinetic competition among elementary steps, forming a continuous kinetic spectrum. It is governed collectively by key factors including steric effects, bond dissociation energies, electronic polarity and solvation effects. This multidimensional regulation inevitably leads to various boundary scenarios and restricts the substrate scope of specific reaction systems. Overall, this kinetic framework demonstrates that terminal selectivity is a complex phenomenon strongly dependent on reaction conditions, and provides new insights for designing precision C(sp^3^)–H functionalization protocols.

Looking ahead, future advances in transforming empirical qualitative models toward predictive reaction design hinge on the establishment of quantitative structure–activity relationships (QSAR) enabled by data-driven methodologies. Rather than regarding steric hindrance as an unparameterized qualitative barrier, future research workflows should prioritize the simultaneous parameterization of multidimensional steric features and intrinsic electronic descriptors. The mathematical combination of Sterimol geometric parameters (*i.e.*, directional widths *B*_1_ and *B*_5_, and length *L*) with Hammett constants (*σ*) allows the construction of high-dimensional free-energy landscapes for reaction systems.^120^ Such quantitative correlations mathematically clarify how electronic properties alter transition-state geometries and magnify proximal steric repulsions,^121^ thereby furnishing a rigorous theoretical basis for rationalizing the kinetic bottlenecks summarized in this review.

To translate these QSAR-derived energetic landscapes into automated predictive platforms, the above kinetic paradigm can be embedded into hybrid machine learning pipelines. A highly efficient strategy involves the integration of low-cost semiempirical quantum mechanics (SQM) with statistical learning algorithms for the rapid assessment of site selectivity in structurally complex molecules. Within such hybrid frameworks, absolute activation energies rapidly computed *via* tight-binding methods (*e.g.*, GFN2-xTB) are further calibrated using molecular fingerprint regressors and physical organic descriptors, which endow the models with reliable extrapolative capacity beyond the interpolation boundaries of training datasets.^122^ Furthermore, to mitigate the data scarcity challenge inherent to profiling intricate C(sp^3^)–H functionalization reactivity, active learning workflows can drive targeted dataset expansion. Guided by uncertainty quantification and structural similarity metrics, these models selectively sample highly informative substrate candidates for experimental validation, substantially reducing the demand for exhaustive high-throughput experimentation (HTE).^123^

Finally, the implementation of machine learning for predictive C–H functionalization necessitates the digitization of catalyst spatial environments *via* shape-agnostic, unbiased structural descriptors.^124,125^ Quantitative mapping of the catalyst first coordination sphere using percent buried volume (%*V*_Bur_) and numerical discretization of topographic steric maps into Cartesian coordinate arrays enables the full structural characterization of asymmetric catalytic pockets as unique digital fingerprints.^124^ These digitized steric matrices, when combined with electrostatic potential distributions, serve as robust input features for deep-learning architectures to support real-time catalyst optimization and high-throughput virtual screening. The synergistic integration of these automated geometric descriptors with the kinetic bottleneck analyses outlined in this review will establish a quantitative, condition-adaptive framework for the design of high-precision catalytic systems toward selective C(sp^3^)–H functionalization.

## Author contributions

J.-L. Tu conceived the review framework and drafted the original manuscript. L. Guo assisted with manuscript revision. B. Huang performed thorough revision to improve the written content and graphical presentations. W. Xia supervised the project and offered critical academic insights. All authors engaged in in-depth discussions on manuscript details, examined and approved the final version of the manuscript.

## Conflicts of interest

There are no conflicts to declare.

## Data Availability

No primary research results, software or code have been included and no new data were generated or analysed as part of this review.
